# Supplementary material for: CD44 connects autophagy decline and ageing in the vascular endothelium
Source: Nat Commun. 2023 Sep 8;14:5524. doi: 10.1038/s41467-023-41346-y (PMC10491636; doi:10.1038/s41467-023-41346-y)
Supplement: Supplementary file 1 — Supplementary Information [file 41467_2023_41346_MOESM1_ESM.pdf]

Supplementary figure 1

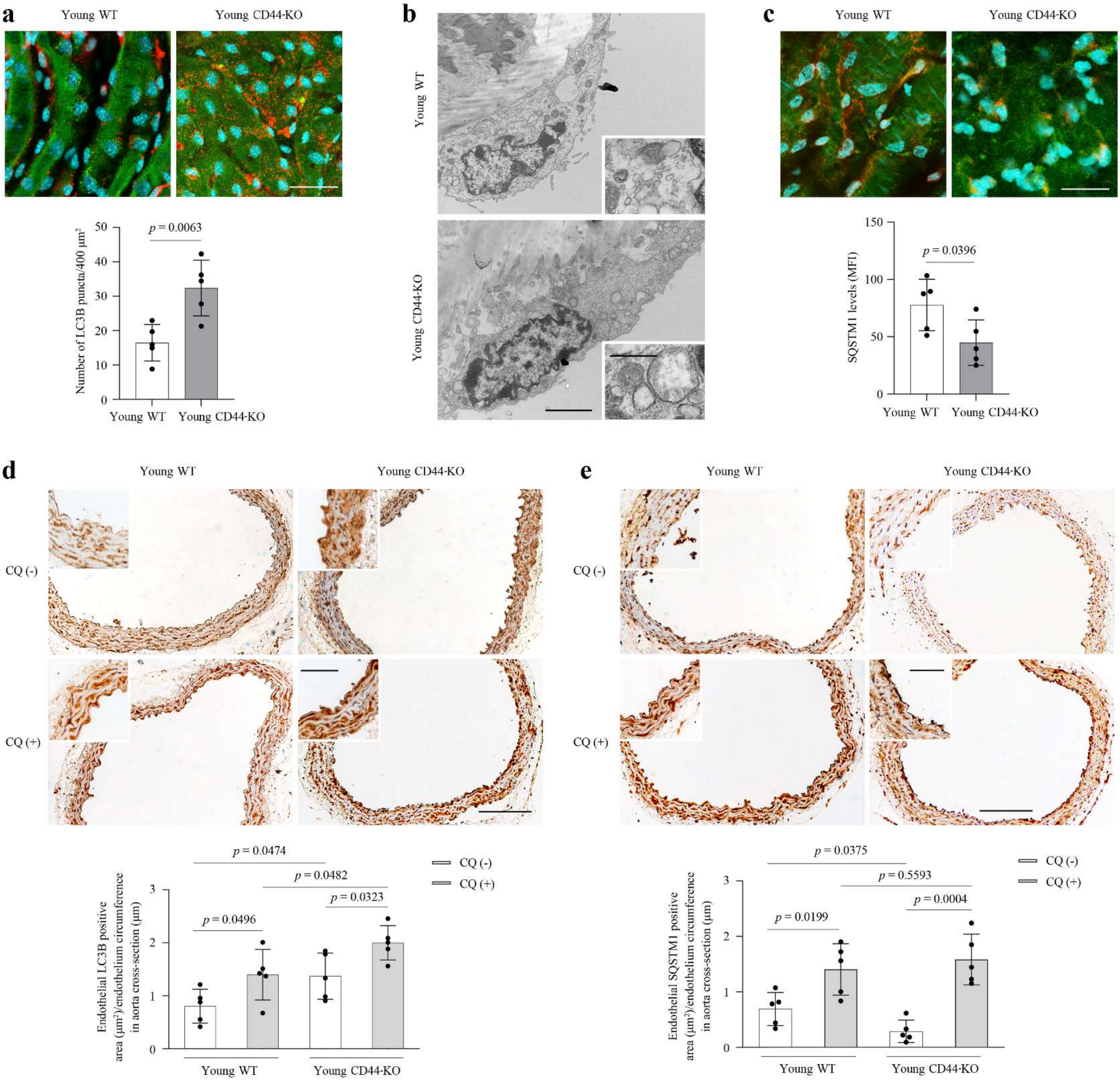

Supplementary figure 1. CD44 knockout increased endothelial autophagic activity in young mice

a,**c**, *En face* staining for LC3B (**a**) or SQSTM1 (**c**) of aortas obtained from WT or

CD44-KO mice (2-4 months). Bar = 50 μm. **b**, Ultrastructural analysis of aortic sections obtained from WT or CD44-KO mice (4 months). Bar = 2 μm. Bar in zoomed figure = 500 nm. **d**,**e**, Immunohistochemical staining for LC3B (**d**) or SQSTM1 (**e**) of aortas obtained from WT or CD44-KO mice (2-4 months) treated with vehicle or CQ by daily intraperitoneal injection (60 mg/kg/day) for 4 days. Bar = 120 μm. Bar in zoomed figure = 50 μm. n = 5 mice per group. Two biologically independent experiments. Data are shown as mean ± s.d.; *P* values are derived from Two-tailed unpaired Student's *t*-tests. Source data are provided as a Source data file.

Supplementary figure 2

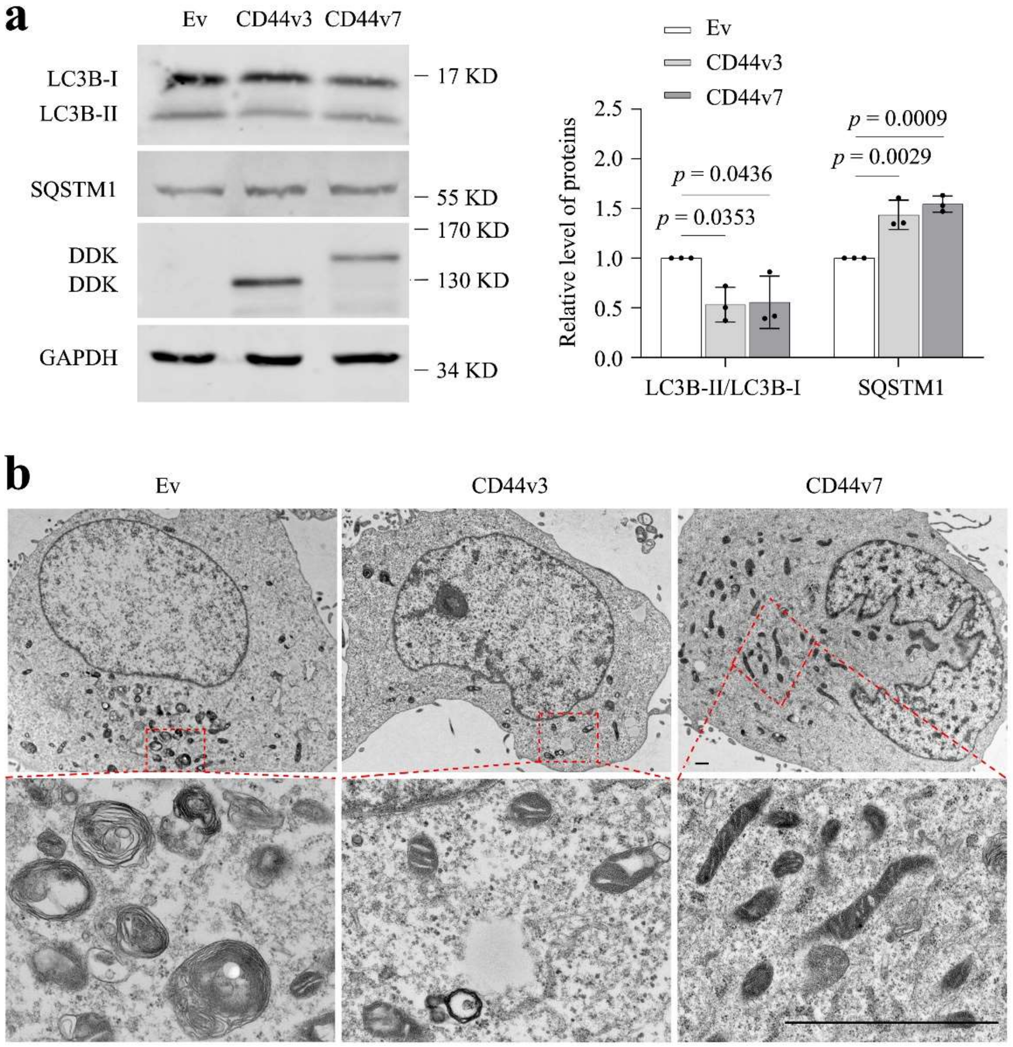

Supplementary figure 2. CD44v3 and CD44v7 reduce autophagy in HUVECs

a, Western blot analysis of LC3B-II/LC3B-I and SQSTM1 in HUVECs (PD3-PD6) transduced with empty vector (Ev), CD44v3 or CD44v7. **b**, Ultrastructural analysis of HUVECs transduced with Ev, CD44v3 or CD44v7 at PD3-PD6. Boxed areas are enlarged below each image. Bar = 1 μm. Three biologically independent experiments. Data are shown as mean ± s.d.; *P* values are derived from one-way ANOVA with Dunnett's multiple comparisons test. Source data are provided as a Source data file.

Supplementary figure 3

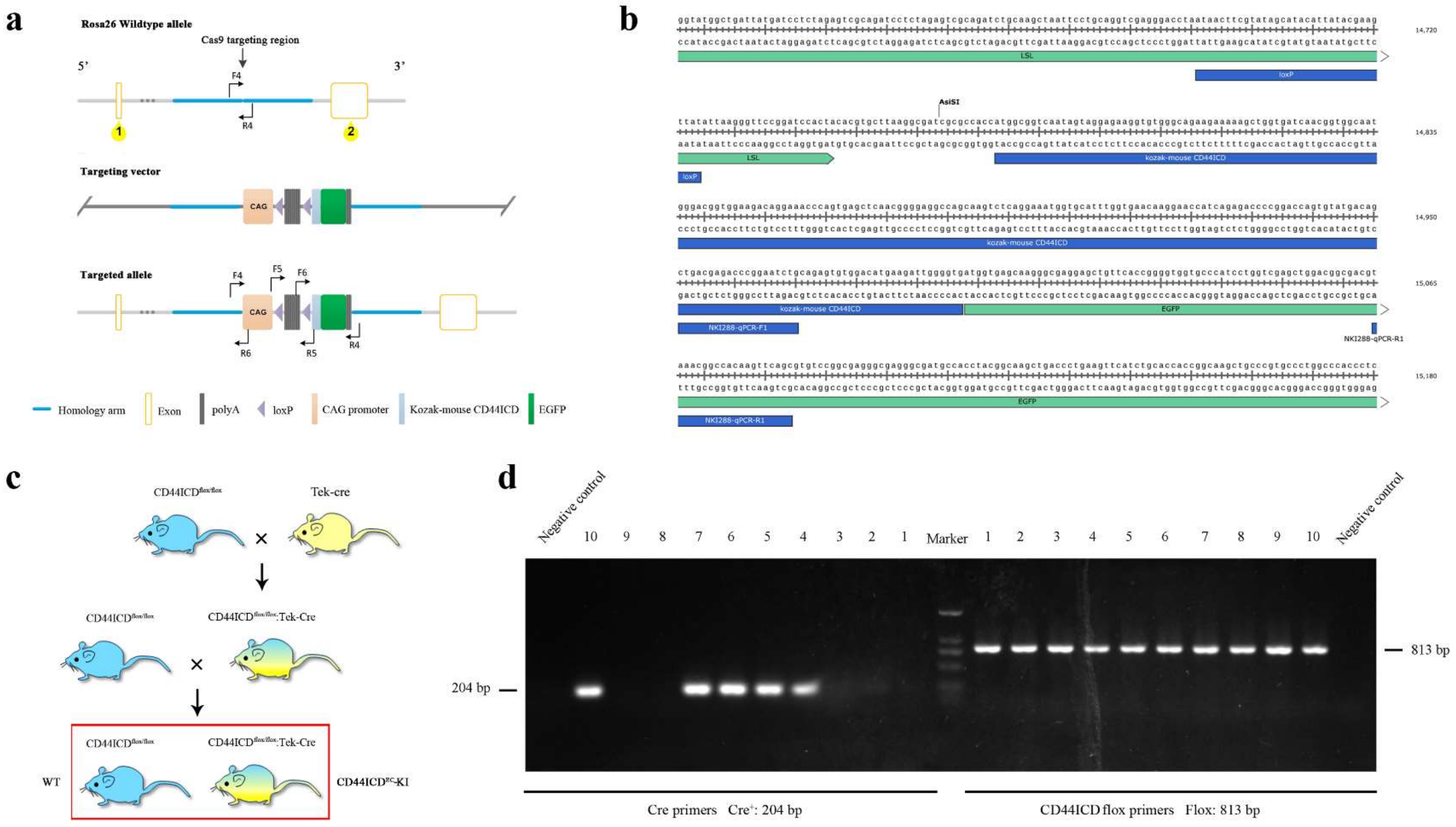

Supplementary figure 3. Generation of CD44ICD^{EC}-KI mice

a, Schematic diagram of the CD44ICD locus and CRISPR-pro-based gene knock-in strategy. **b**, The base sequence of the inserted CD44ICD. **c**, Reproductive strategy diagram of CD44ICD^{EC}-KI mice and their control littermates (WT). **d**, PCR genotyping results of the offspring of WT (CD44ICD$^{flox/flox}$) mice and CD44ICD^{EC} KI (CD44ICD$^{flox/flox}$:Tek-cre) mice by PCR (FW primer 5'-CAGACTTGTGGGATACAGAAGAC-3' and RW primer 5'-AGATGTACTGCCAAGTAGGAAAGTC-3') and (FW primer 5'-GAACGCACTGATTTCGACCA-3' and RW primer 5'-

GCTAACCAGCGTTTTCGTTC-3’). 1-3, 8 and 9 are WT mice, 4-7 and 10 are CD44ICD^{EC} KI (CD44ICD$^{flox/flox}$:Tek-Cre) mice.

Supplementary figure 4

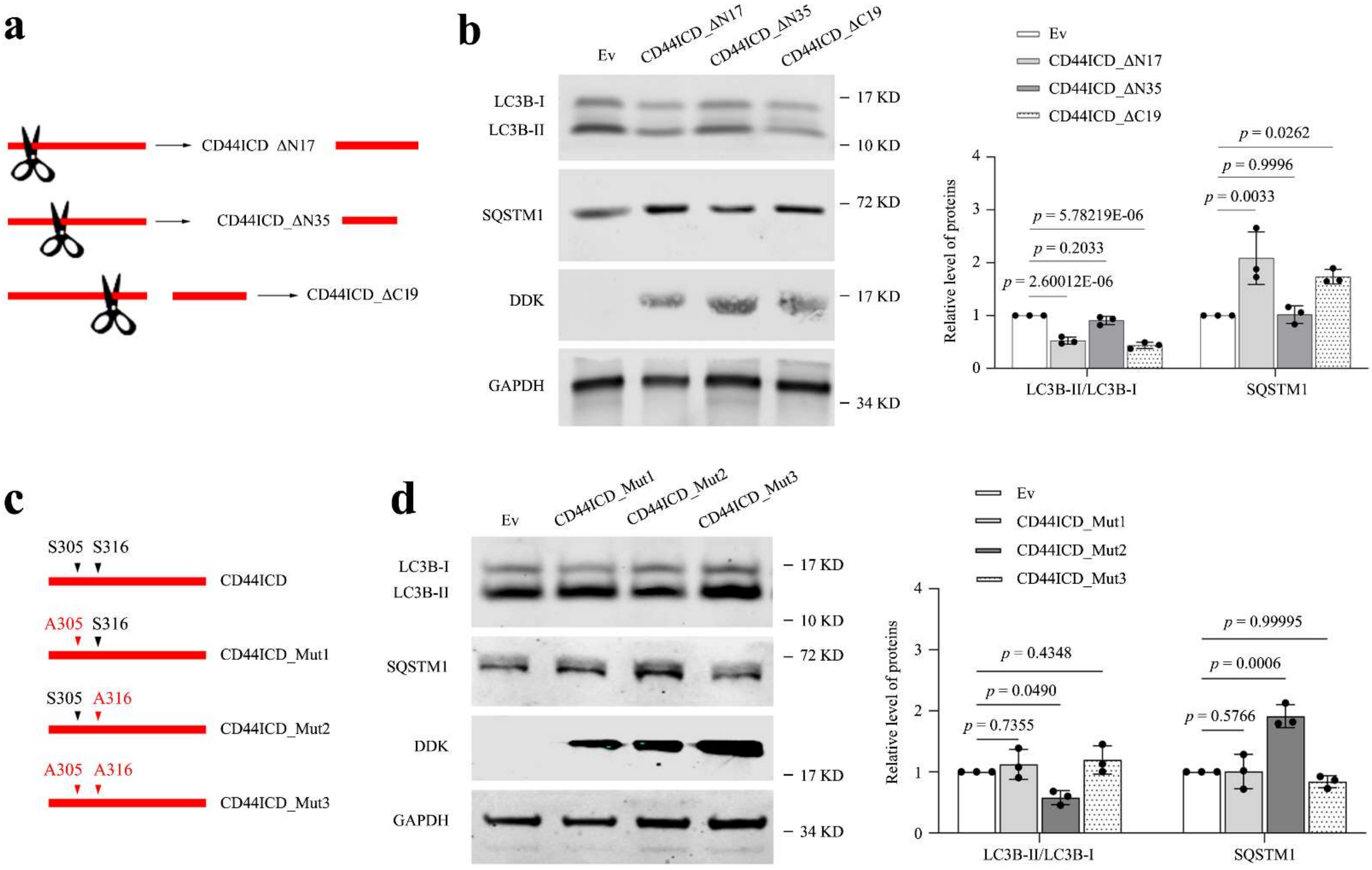

Supplementary figure 4. 18-amino acid (S^{305}-A^{322}) stretch of CD44ICD and the phosphorylation at S305 is needed for reducing autophagy

a, Schematic of the CD44ICD truncated mutants used in the study. **b**, Western blot analysis of LC3B-II/LC3B-I and SQSTM1 in HUVECs (PD3-PD6) transduced with Ev, CD44ICD_ΔN17, CD44ICD_ΔN35 or CD44ICD_ΔC19. **c**, Schematic of the CD44ICD phosphorylation site S305/S316 mutants used in the study. Serine-to-alanine mutation at position 305 or/and S316. **d**, Western blot analysis of LC3B-II/LC3B-I and SQSTM1 in HUVECs (PD3-PD6) transduced with Ev, CD44ICD_Mut1, CD44ICD_Mut2 or CD44ICD_Mut3. Three biologically independent experiments.

Data are shown as mean ± s.d.; *P* values are derived from one-way ANOVA with Dunnett's multiple comparisons test. Source data are provided as a Source data file.

Supplementary figure 5

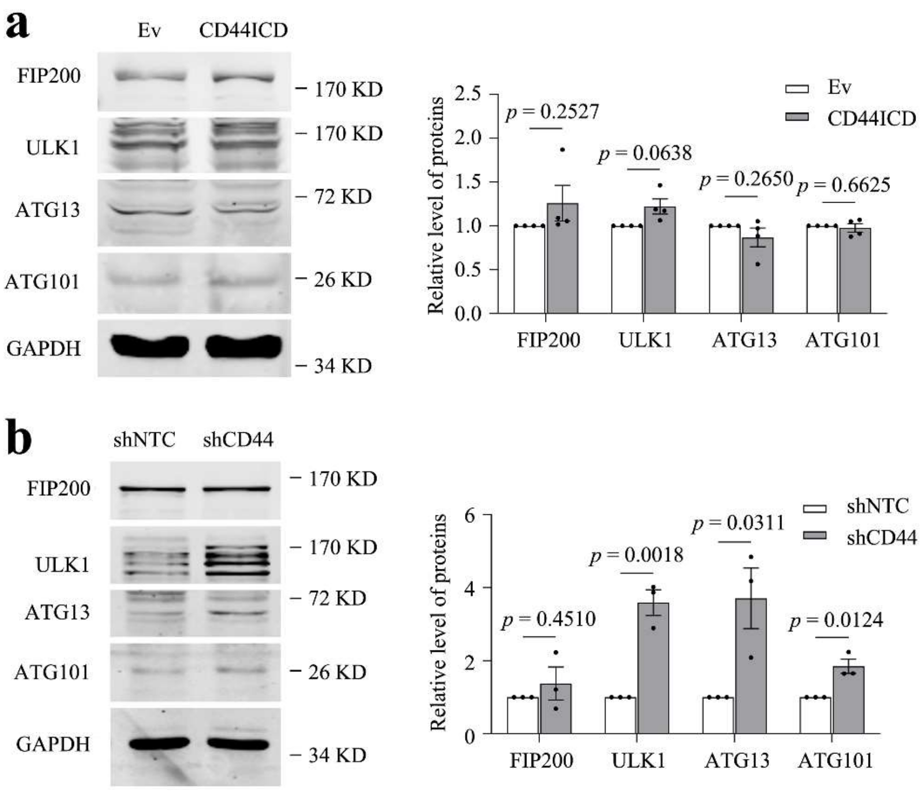

Supplementary figure 5. The effects of CD44 and CD44ICD on the levels of the core proteins of the ULK1 complexes

a,**b**, Western blot analysis of the core proteins of the ULK1 complexes in HUVECs (PD3-PD6) transduced with Ev, CD44 and CD44ICD (**a**) or expressing non-targeting control shRNA (shNTC) or shCD44 (**b**). Three (**b**) or four (**a**) biologically independent experiments. Data are shown as mean ± s.d.; *P* values are derived from Two-tailed unpaired Student's *t*-tests. Source data are provided as a Source data file.

Supplementary figure 6

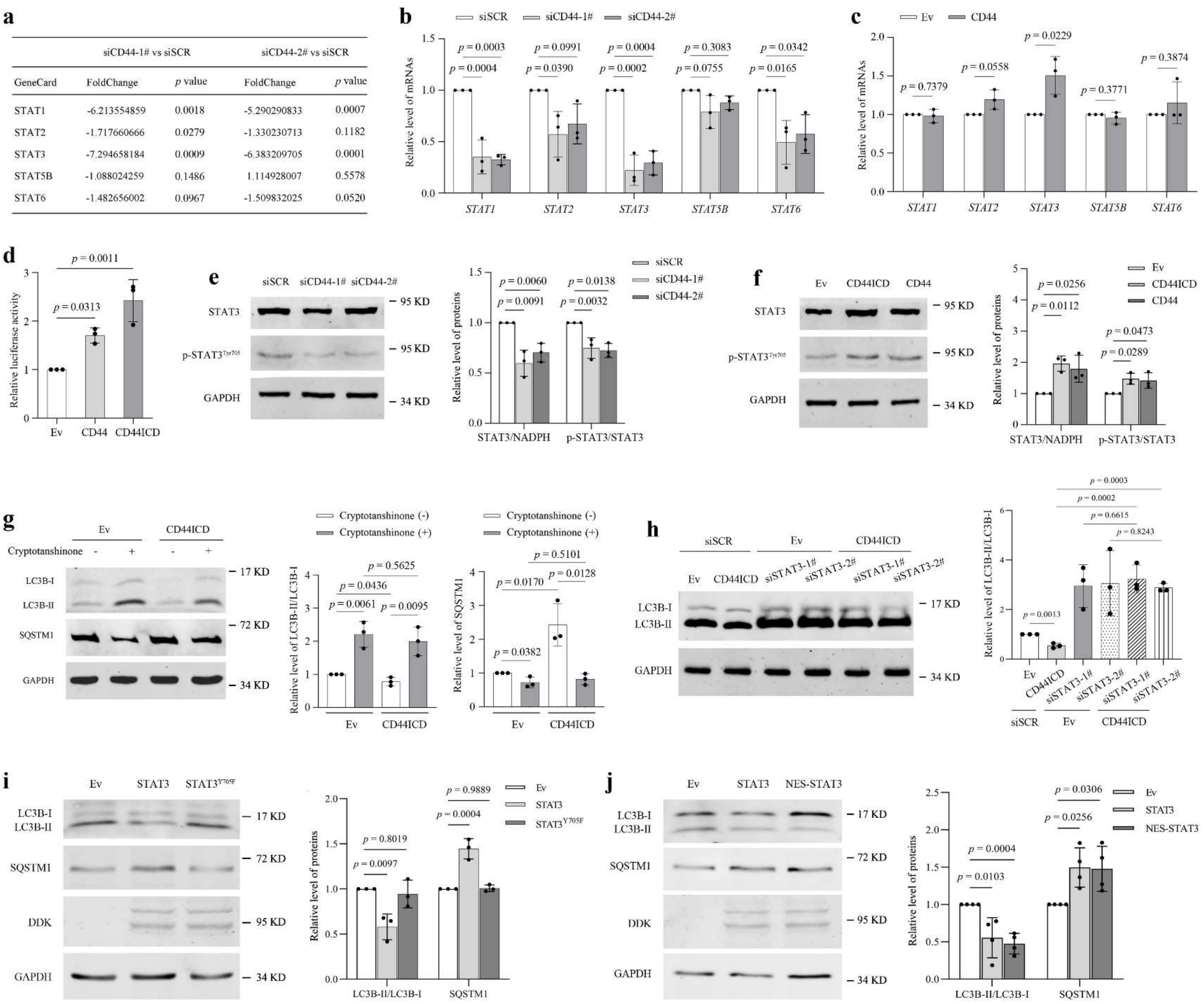

GeneCard	siCD44-1# vs siSCR FoldChange	siCD44-1# vs siSCR *p* value	siCD44-2# vs siSCR FoldChange	siCD44-2# vs siSCR *p* value
STAT1	-6.213554859	0.0018	-5.290290833	0.0007
STAT2	-1.717660666	0.0279	-1.330230713	0.1182
STAT3	-7.294658184	0.0009	-6.383209705	0.0001
STAT5B	-1.088024259	0.1486	1.114928007	0.5578
STAT6	-1.482656002	0.0967	-1.509832025	0.0520

Supplementary figure 6. CD44 decreases autophagy through STAT3

a, The table shows the altered expression of STAT genes in HUVECs transfected with CD44 siRNAs using microarray analysis. **b**,**c**, qRT-PCR analysis of STATs levels in HUVECs (PD3-PD6) transfected with scrambled siRNA and CD44 siRNAs (**b**), or Ev and CD44 (**c**). **d**, Dual luciferase gene reporter assay of STAT3 transcriptional activity

in HUVECs (PD3-PD6) transduced with Ev, CD44 or CD44ICD. **e**,**f**, Western blot analysis of STAT3 and p-STAT3 in HUVECs (PD3-PD6) transfected with scrambled siRNA and CD44 siRNAs (**e**), or Ev, CD44 and CD44ICD (**f**). **g**, Western blot analysis of LC3B-II/LC3B-I in HUVECs (PD3-PD6) transduced with Ev or CD44ICD in the presence or absence of cryptotanshinone (2 μM) for 18 h. **h**, Western blot analysis of LC3B-II/LC3B-I and SQSTM1 in HUVECs (PD3-PD6) co-transfected with STAT3 siRNAs and CD44ICD. **i**,**j**, Western blot analysis of LC3B-II/LC3B-I and SQSTM1 in HUVECs (PD3-PD6) transfected with Ev, STAT3 and STAT3^{Y705F} (**i**), or Ev, STAT3 and NES-STAT3 (**j**). Three (**a**-**i**) or four (**j**) biologically independent experiments. Data are shown as mean ± s.d.; *P* values are derived from one-way ANOVA with Dunnett's multiple comparisons test (**a**, **b**, **d**, **e**, **f**, **i**, **j**), Two-tailed unpaired Student's *t*-tests (**c** and **g**), one-way ANOVA with Dunnett's multiple comparisons test and Two-tailed unpaired Student's *t*-tests (**h**). Source data are provided as a Source data file.

Supplementary figure 7

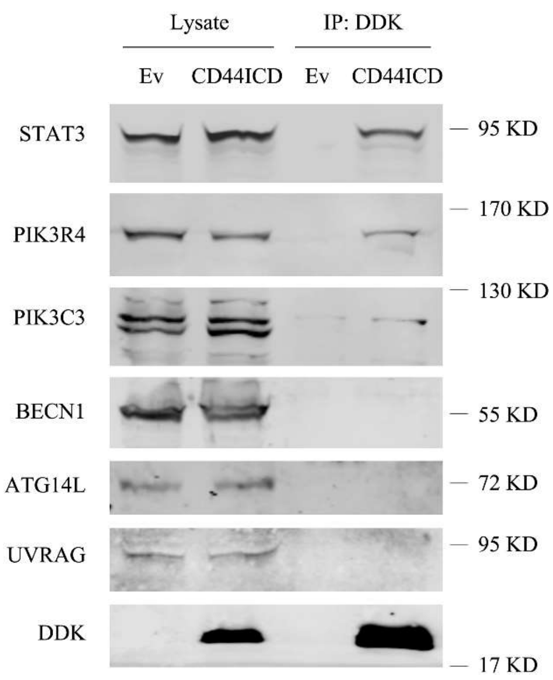

Supplementary figure 7. CD44ICD coimmunoprecipitated with STAT3, PIK3C3 and PIK3R4 but not with BECN1, Atg14L and UVRAG

Western blots showing co-IP of CD44ICD with endogenous STAT3 and core proteins of the PtdIns3K complexes in HUVECs transduced with Ev or CD44ICD (DDK tag). Lysates, whole cell lysates; IP, immunoprecipitates. Three biologically independent experiments.

Supplementary figure 8

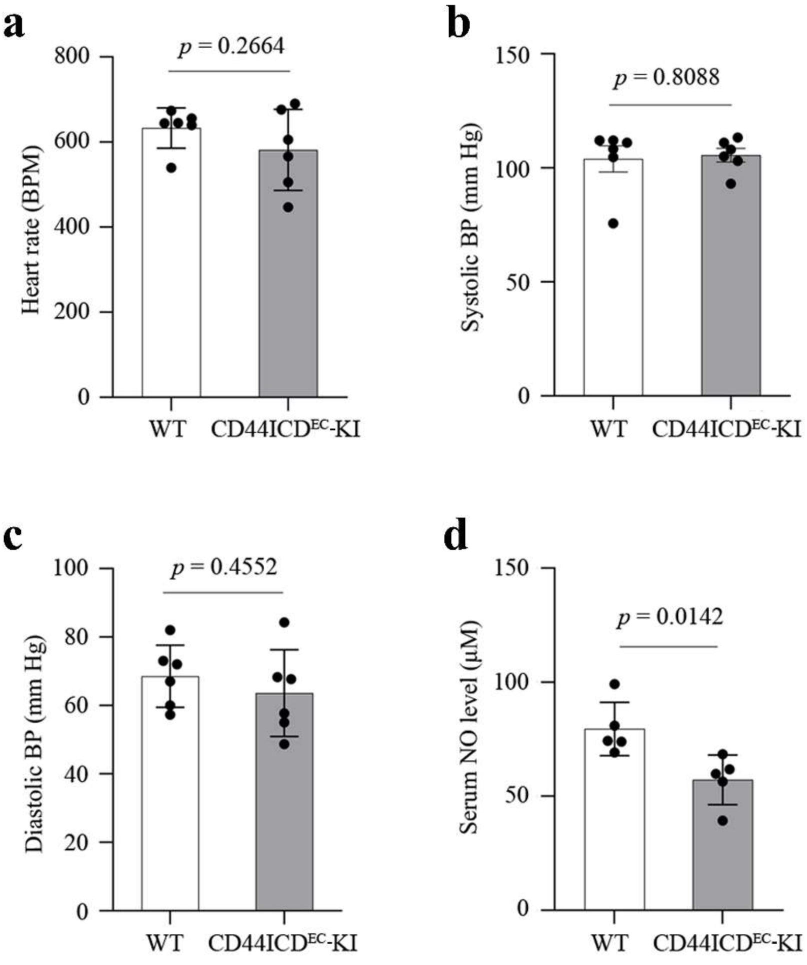

Supplementary figure 8. Effects of endothelium-specific overexpression of CD44ICD on heart rate, blood pressure (BP) and serum NO levels in mice

a,**b**,**c**,**d**, Heart rate (**a**), systolic (**b**), BP (**c**), and serum NO levels (**d**) in WT (CD44ICD$^{flox/flox}$) and CD44ICD^{EC} KI (CD44ICD$^{flox/flox}$:Tek-Cre) mice (8-10 months). BPM, beats per miniute. For **a-c**, n = 6 mice per group. For **d**, n = 5 mice per group. Two biologically independent experiments. Data are shown as mean ± s.d.; *P* values are derived from Two-tailed unpaired Student's *t*-tests. Source data are provided as a Source data file.

Supplementary figure 9

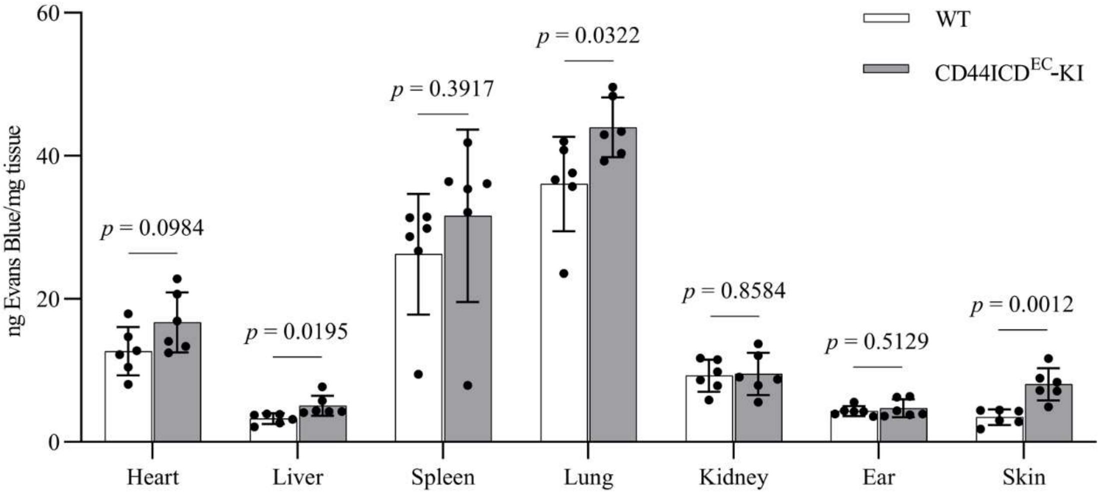

Supplementary figure 9. Basal vascular permeability

The tissues were obtained from WT and CD44ICD^{EC} KI mice (14-17 months). n = 6 mice per group. Two biologically independent experiments. Data are shown as mean ± s.d.; *P* values are derived from Two-tailed unpaired Student's *t*-tests. Source data are provided as a Source data file.

Supplementary figure 10

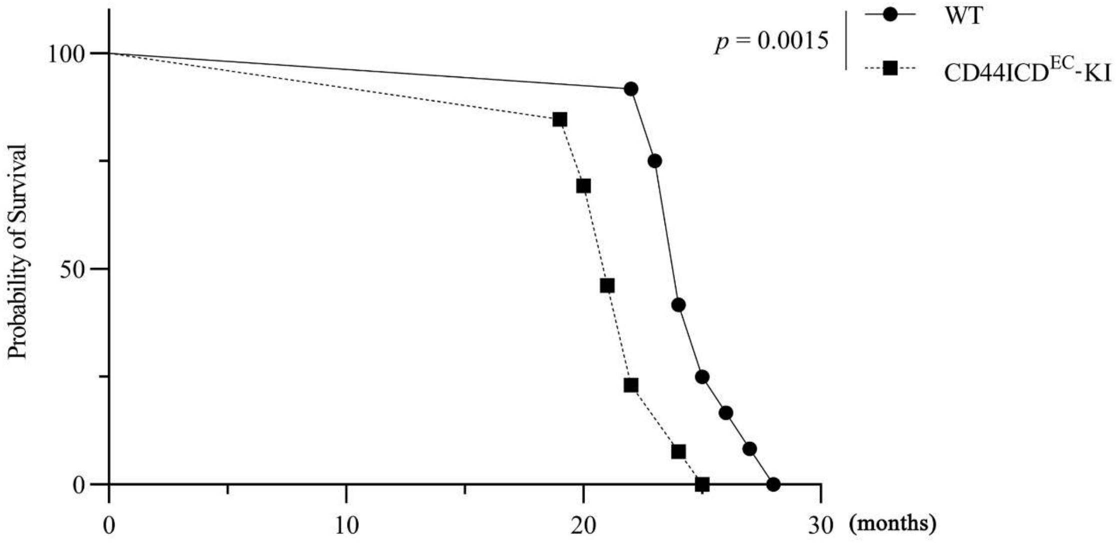

Supplementary figure 10. Endothelium-specific overexpression of CD44ICD significantly shortened the lifespan of mice.

Survival curves of WT and CD44ICD^{EC}-KI mice. n = 12 (WT) mice, n = 13 (CD44ICD^{EC}-KI) mice. Two biologically independent experiments. *P* values are derived from Log-rank (Mantel-Cox) test. Source data are provided as a Source data file.

Supplementary figure 11

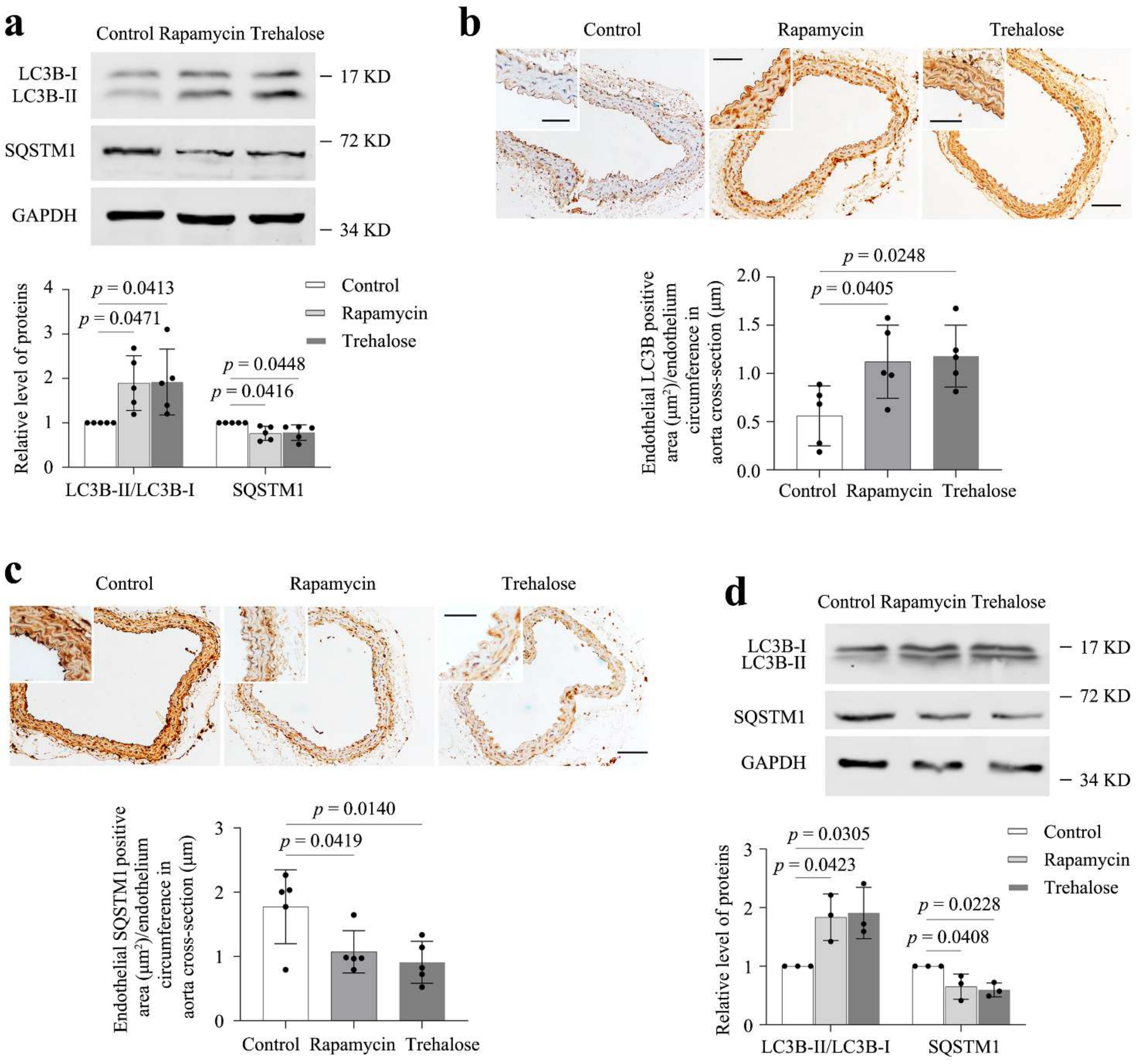

Supplementary figure 11. Rapamycin or trehalose treatment activates autophagy in CD44ICD^{EC}-KI mice and CD44ICD-overexpressing HUVECs

a,**b**,**c**, Western blot analysis of LC3B-II/LC3B-I and SQSTM1 (**a**) or immunohistochemical staining for LC3B (**b**) or SQSTM1 (**c**) of aortas obtained from CD44ICD^{EC}-KI mice (8 months) received intraperitoneal injection of vehicle and rapamycin once every 2 days for a total of 6 injections (4 mg/kg/day), or fed with

trehalose for 25 days (administration of 2% trehalose in drinking water). Bar = 200 μm. Bar in zoomed figure = 100 μm. **d**, Western blot analysis of LC3B-II/LC3B-I and SQSTM1 in CD44ICD-overexpressing HUVECs (PD2) treated with rapamycin (1 nM) or trehalose (100 μM) for 15 days. For **a**, **b**, **c**, n = 5 mice per group. Two (**a**, **b**, **c**) or three (**d**) biologically independent experiments. Data are shown as mean ± s.d.; *P* values are derived from one-way ANOVA with Dunnett's multiple comparisons test. Source data are provided as a Source data file.

Supplementary figure 12

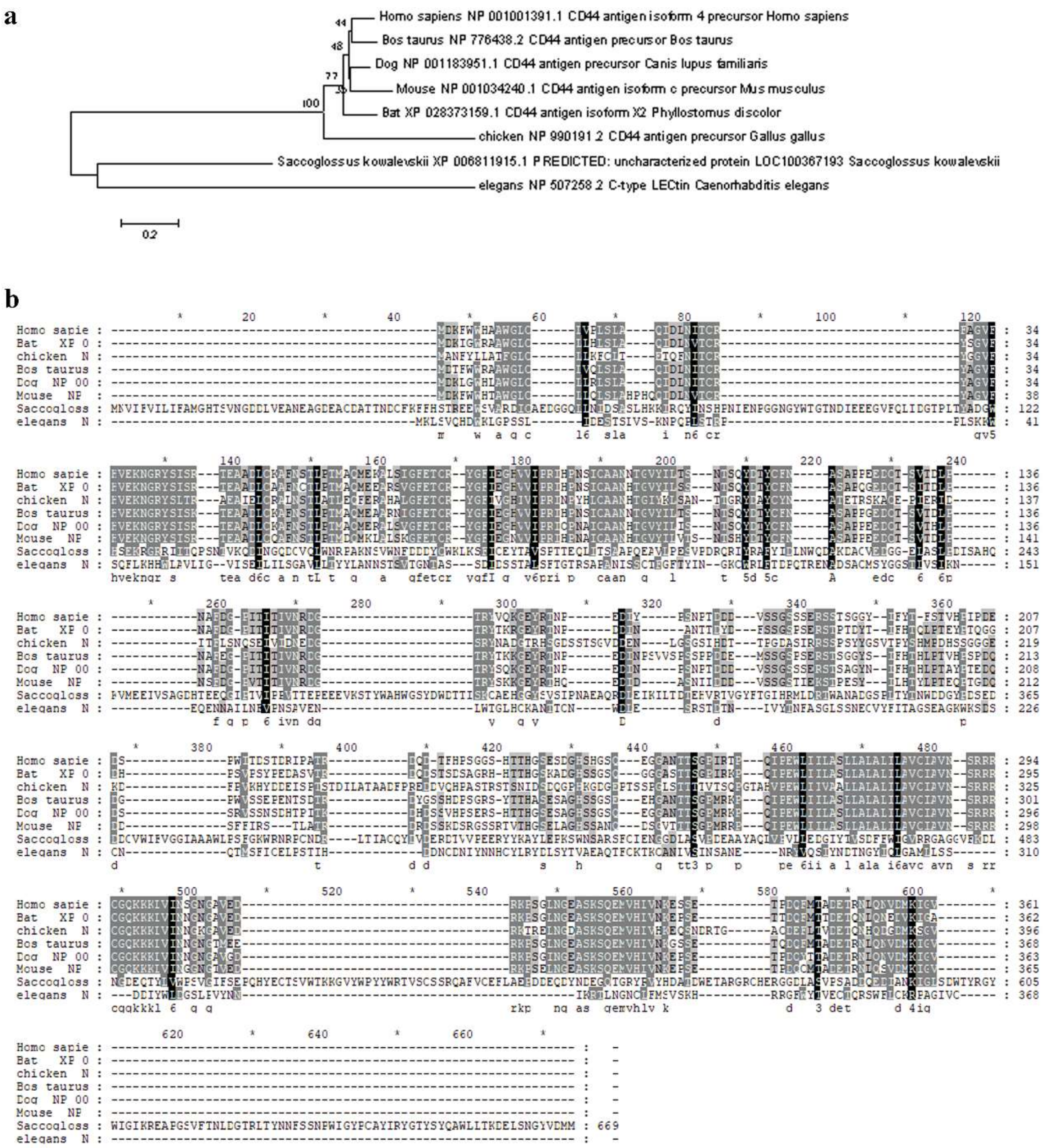

Supplementary figure 12. Identification of *C. elegans* CD44 homolog

a, Phylogenetic tree generated by ClustalW analysis showing that NP_507258.2 is similar to Homo sapiens NP_001001391.1, and Mouse NP_001034240. **b**, Amino-acid alignment of the C-terminal regions of Rubicon homologs from several species (Homo sapiens; Bat; Chicken; Bos taurus; Dog; Mouse; Saccoglossus kowalevskii; Elegans)

Supplementary figure 13

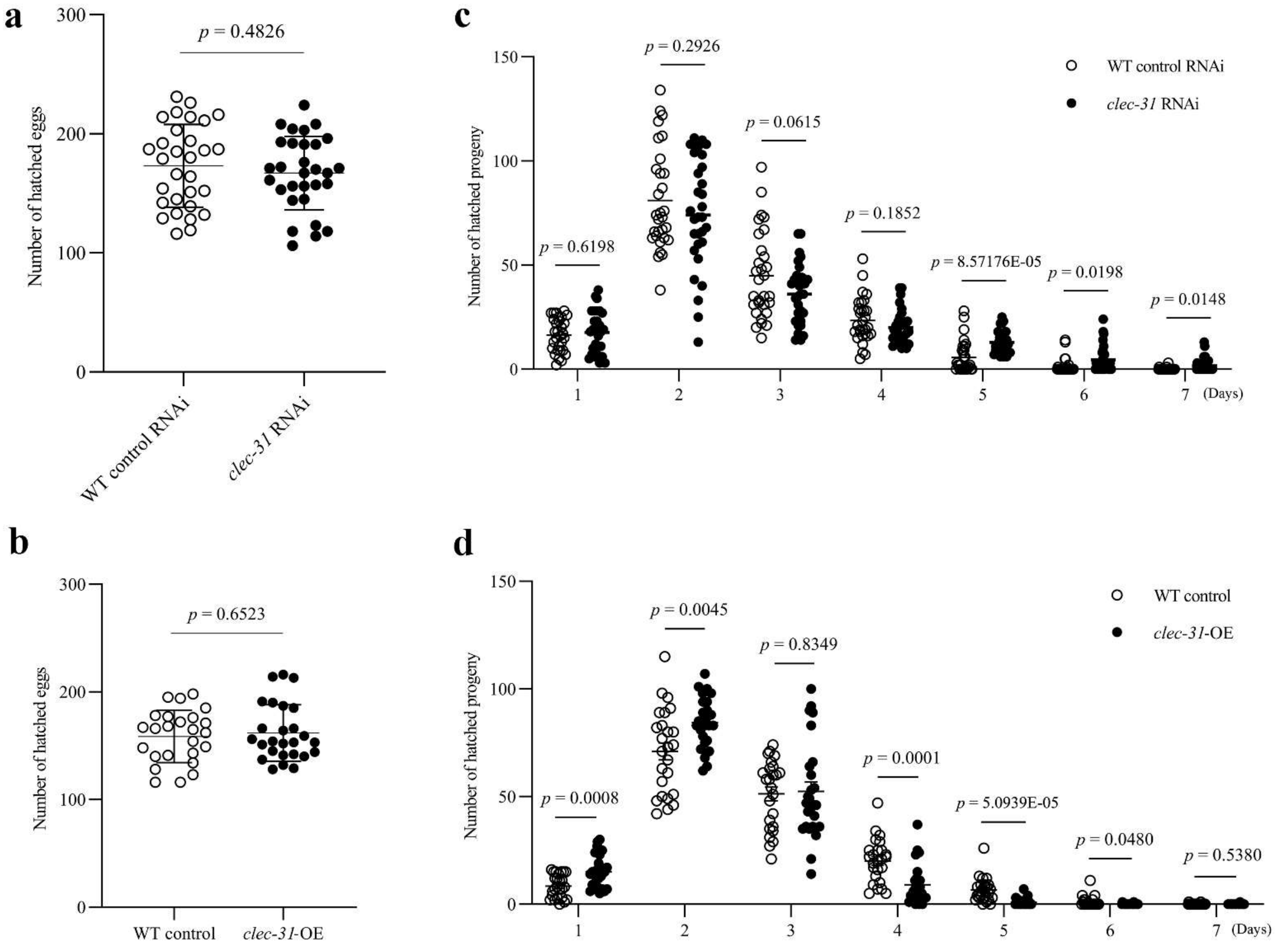

Supplementary figure 13. The changes of clec-31 level alter the reproductive period of nematodes with no significant change in total number of hatched eggs

a,**b**, Knockdown (**a**) or overexpression (**b**) of *clec-31*does not significantly change total number of hatched eggs. **c**,**d**, Reproductive analysis of *clec-31*-knockdown nematodes (**c**) and *clec-31*-OE nematodes (**d**). For **a** and **c**, n = 30 nematodes per group. For **b** and **d**, n = 25 (WT control) nematodes, n = 26 (*clec-31*-OE) nematodes. Three biologically independent experiments. Data are shown as mean ± s.d.; *P* values are derived from Two-tailed unpaired Student's *t*-tests. Source data are provided as a Source data file.

Supplementary figure 14

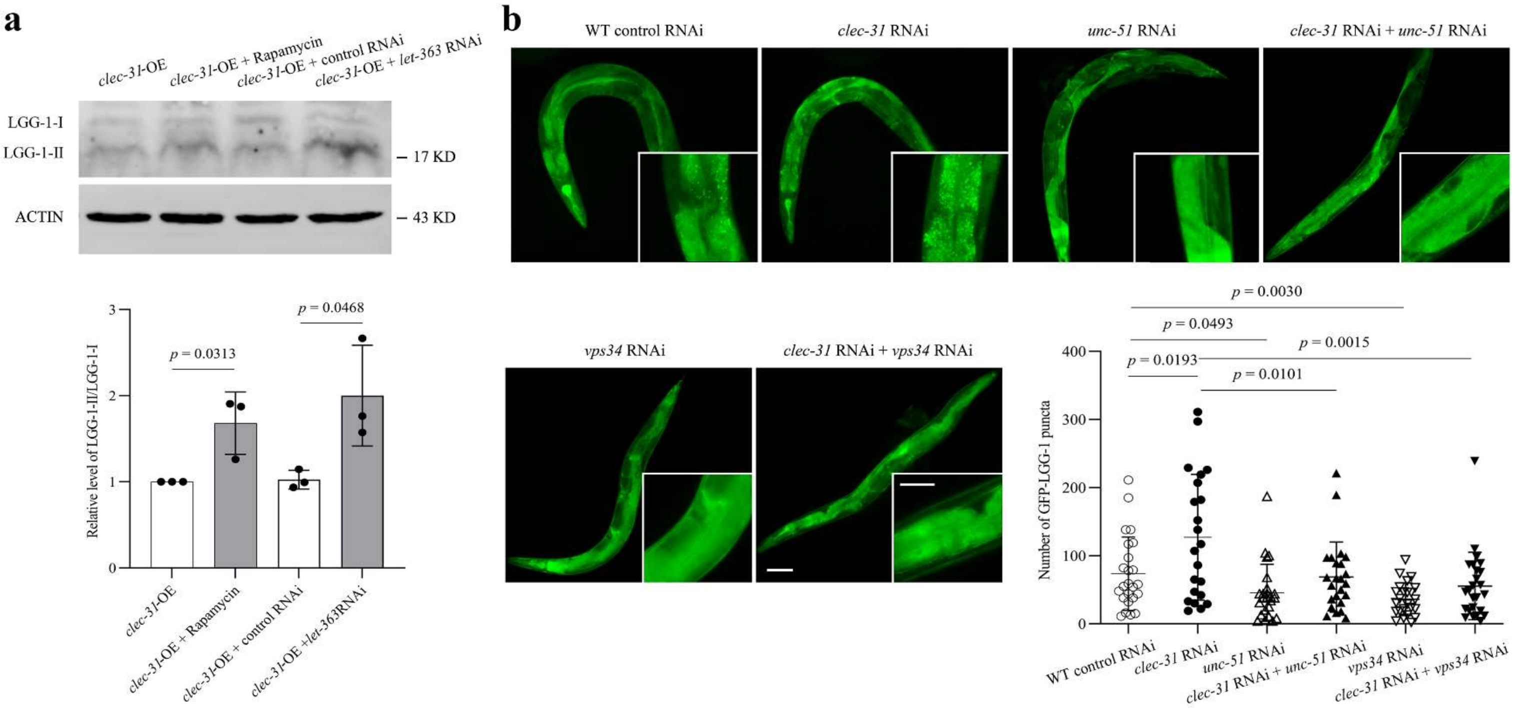

Supplementary figure 14. Rapamycin treatment or knockdown of *let-363* activates autophagy in *clec-31*-OE nematodes, and knockdown of autophagy genes suppressed autophagic activity in *clec-31*-knockdown nematodes

a, Western blot analysis of LGG-1-II/LGG-1-I in *clec-31*-OE nematodes treated with rapamycin or fed RNAi bacteria targeting empty vector or *let-363*. **b**, Detection of GFP::LGG1 puncta in nematodes fed RNAi bacteria targeting empty vector, *clec-31*, *unc-51* or *vps34*, alone or in combination. Bar = 100 µm. Bar in zoomed figure = 50 µm. For **b**, n = 24 (WT control RNAi) nematodes, n = 22 (*clec-31* RNAi) nematodes, n = 24 (*unc-51* RNAi) nematodes, n = 24 (*clec-31* RNAi+ *unc-51* RNAi) nematodes, n = 24 (*vps34* RNAi) nematodes, n = 25 (*clec-31* RNAi+ *vps34* RNAi) nematodes. Three biologically independent experiments. Data are shown as mean ± s.d.; *P* values are derived from Two-tailed unpaired Student's *t*-tests. Source data are provided as a Source data file.

Supplementary figure 15

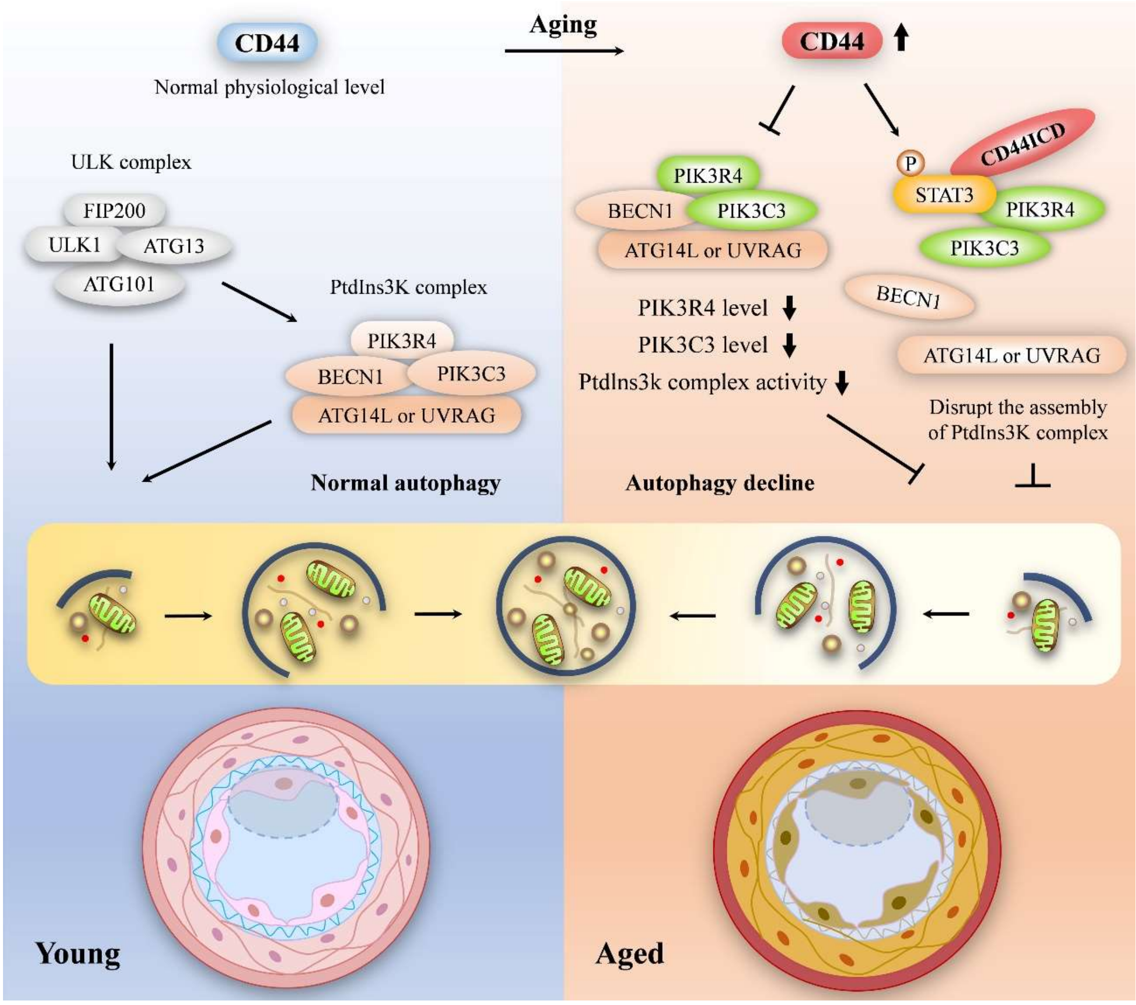

Supplementary figure 15. Model for the role of CD44 connects autophagy decline and ageing in VECs

In young VECs with normal basal autophagy, CD44 has a low expression, which maintains the "young status" of the cells. CD44 increases with age, which suppresses the levels of PIK3R4 and PIK3C3, the core components of the PtdIns3K complex and the activity of PIK3C3 kinase. In addition, CD44ICD binds PIK3R4 and PIK3C3 *via* binding and activating STAT3. As a result, PIK3R4 and PIK3C3 are lost in the PtdIns3K complex, giving rise to decreased autophagy and senescence of VECs.

Supplementary table 1 Detailed list of materials used in this study

ANTIBODIES	SOURCE	IDENTIFIER
CD44,1:1000/1:1000	Cell Signaling Technology	Cat#5640 (M)Cat#3578(R)
CDKN2A,1:1000/1：500	Abcam/ ABclonal	Cat#AB51243/Cat#A11058
CDKN1A,1:1000/1:500/1:1000	Abcam/ ABclonal/ Cell Signaling	Cat#AB109199/Cat#A1483/ Cat#37543
LC3,1:15000	SIGMA	Cat#SAB1305552
LC3,1:1000	SIGMA	Cat#L7543
LC3,1:1000	Cell Signaling Technology	Cat#12741S
LC3,1:1000	Proteintech	Cat#14600-1-AP
p-eNOS,1;500	ABclonal	Cat#AP0515
SQSTM1,1:15000	SIGMA	Cat#P0067
Flag (DDK),1:1000	OriGene	Cat#TA100023
Flag,1:2000	Proteintech	Cat#20543-1-AP
Flag,1:1000	Cell Signaling Technology	Cat#14793
PIK3R4,1:1000	SIGMA	Cat#HPA036032
PIK3C3,1:1000	SIGMA	Cat#V9764
Atg14L,1:1000	Cell Signaling Technology	Cat#96752
Beclin1,1:1000/1:2000	SIGMA/bioworld	Cat#B6061/Cat#AP6020
UVRAG,1:1000/1:1000/1:1000	SIGMA/Cell Signaling Technology/Cell Signaling Technology	Cat#SAB4200005/Cat#13115/Cat#5320
STAT3,1:1000/1:1000	Cell Signaling Technology	Cat#9139/Cat#12640
p-STAT3,1:1:2000	Cell Signaling Technology	Cat#9145
GAPDH,1:1000	SIGMA	Cat#G9549
ULK1,1:1000	ABclonal	Cat#A8529
ATG13,1:1000	ABclonal	Cat#A0690
ATG101,1:1000	Proteintech	Cat#26562-1-AP
FIP200,1:1000	ABclonal	Cat#A14685
P-STAT1,1:1000	ABclonal	Cat#AP0054
STAT1,1:1000	ABclonal	Cat#A12075
CD31,1:500	Santa cruz	Cat#sc-376764
DDDDK-Tag,1:200	ABclonal	Cat#AE005

REAGENTS	SOURCE	IDENTIFIER
Yeast Nitrogen Base,without Amino Acids	BBI Life Sciences Corporation	Cat#A610507-0100
DO Supplement-Ade/-His/-Leu/-Trp	TaKaLa	Cat#630428
Poly(ethylene glycol) 3350	Solarbio	Cat#P8040

3-AT	BBI Life Sciences Corporation	Cat#A601149
T4 DNA Ligase	TaKaLa	Cat#2011A
X-α-gal	Solarbio	Cat#B8050
DAPI	SIGMA	Cat#28718-90-3
Albumin(bovine fraction V)	Meilunbio	Cat#MB4219
Cholesterol	Diamond	Cat#A100433
Chloroquine diphosphate salt	SIGMA	Cat#C6628
Gelatin from bovine skin	SIGMA	Cat#G9391
ChamQ Universal SYBR qPCR Master Mix	Vazyme	Cat#Q711-02
Collagenase, Type 1	Diamond	Cat#A004194
D-(+)-Trehalose dihydrate	BBI Life Sciences Corporation	Cat#A600966
Puromycin Dihydrochloride	Beyotime	Cat#ST551
Lipofectamine RNAiMAX Reagent	Invitrogen	Cat#13778-150
Lipofectactamine 3000 Transfection Kit	Invitrogen	Cat#L3000-001
Opti-MEMTM	Gibco	Cat#31985-070
EBSS	Gibco	Cat#24010-043
Polybrene (Hexadimethrine Bromide)	Beyotime	Cat#C0351
Rapamycin(Sirolimus)	APExBIO	Cat#A8167
3-Methyladenine	APExBIO	Cat#A8353
Bafilomycin A1	MCE	Cat#HY-100558
Penicillin-Streptomycin Liquid	Solarbio	Cat#P1400
Fetal bovine serum (FBS)	EVERY GREEN	Cat#11011-8611
StealthTM RNAi Negative Control Medium GC Duplex	Invitrogen	Cat#2263012
Stat3 siRNA-1#	Invitrogen	Cat#6679727
CD44 siRNA-1#	Invitrogen	Cat#7335065
DMEM High Glucose	VivaCell BIOSCIENCES	Cat#C3110-0500
Endothelial Cell Medium	ScienCell	Cat#1001
NEW BORNOALF SERUM Collected and Processed in New Zealand	Cytiva	Cat#SH30401.07
HiScript II Q RT SuperMix for qPCR	Vazyme	Cat#R223
TritonX-100	Solarbio	Cat#T8200

Coomassie brilliant blue G-250	Solarbio	Cat#C8420
Gama-secretase inhibitor(GSI)	Sigma	Cat#S2188
Polyethylenimine	Polysciences	Cat#23966-1
Masson Tricolor Staining Kit	Servicebio	Cat#G1006
Resorcinol staining solution	Servicebio	Cat#G1054
H&E Staining Kit (Hematoxylin and Eosin)	Servicebio	Cat#G1005
RaPure Total RNA Kit	Magen	Cat#R4011-02
NGzol RNA Uptake Kit	HLINGENE	Cat#NG304S
Senescence β-Galactosidase Staining Kit	Beyotime	Cat#C0602
Yeast Plasmid Preps Kit	Coolaber	Cat#PE051
EndoFree Plamisid Midi Kit	CWBIO	Cat#CW2105S
SanPrep Column PCR Product Purification Kit	Sangon Biotech	Cat#B518141-0100
Gel Extraction Kit	CWBIO	Cat#CW2302M
Nitric Oxide (NO) assay kit	Nanjing Jiancheng Bioengineering Institute	Cat#A012
GST-tag Protein Purification Kit	Beyotime	Cat#P2262
Dual Luciferase Reporter Gene Assay Kit	Beyotime	Cat#RG027
Evans Blue	Servicebio	Cat#E8010
Formamide	Macklin	Cat#F809511
ATP	Beyotime	Cat#D7378
Chloral hydrate	SIGMA	Cat#23100
Class III PI3-K ELISA Kit	Echelon Biosciences	Cat#K-3000
**CELLS**	**SOURCE**	**IDENTIFIER**
HEK293T cells	ATCC	Cat#CBP60439
HUVECs	In our lab	N/A
HT115(DE3)	Shanghai Weidi Biotechnology	Cat#EC2010
AH109	Shanghai Weidi Biotechnology	Cat#YC1010
DH-5α	Shanghai Weidi Biotechnology	Cat#DL1001
BL21(DE3)	Shanghai Weidi Biotechnology	Cat#EC1002

**ANIMALS**	**SOURCE**	**IDENTIFIER**
Mouse:B6J.Cg-Gt(ROSA)26Sor$^{em16(CAG-Cd44icd)}$/J	Cyagen	Project No: SO171215JW2
Mouse:B6.Cg-Tg(Tek-cre)12Flv/J	Cyagen	Stock No:C001001
Mouse:B6.129(Cg)-Cd44^{tm1Hbg}/J	Jackson Laboratories	Stock No: 005085
Mouse: WT C57BL6/J	Beijing Vital River Laboratory Animal Technology	No.11400700255206
Caenorhabditis elegans:N2（WT）	Professor Ding Chunbang from Sichuan Agricultural University，China	N/A
Caenorhabditis elegans:DA2123:adls2122(lgg-1p::gfp::lgg-1)	Dr Zhang Hong from Institute of Biophysics，Chinese Academy of Sciences，China	N/A
Caenorhabditis elegans:*CLEC*-31 OE Ex [Peft-3::clec-31::unc-54 3'UTR, Psur-5::sur-5::NLSGFP]	SunyBiotech	Project No:ZLUO1-02
**GENECHIPS**	**SOURCE**	**IDENTIFIER**
Affimatrix Genechip	Shanghai Baygene Biotechnologies Company Limited	Project No:HNDYDX001010
**PLASMIDS**	**SOURCE**	**IDENTIFIER**
pHAGE-CMV-MCS-PGK-3×FLAG	Our lab	N/A
psPAX2	Addgene	Cat#12260
pMD2.G	Addgene	Cat#12259
pLKO.1-TRC Cloning Vector	Addgene	Cat#10878
lentiCRISPR v2	Addgene	Cat#52961
pBiFC-VN155	Addgene	Cat#27097
pBiFC-VC155	Addgene	Cat#22011
pGADT7	Addgene	Cat#61702
pGBKT7	Addgene	Cat#61703
pGEX-4T-3	Our lab	N/A
L4440	Addgene	Cat#1654
AdPlus-mCherry-GFP-LC3B	Beyotime	Cat#C3012

Ad-GFP-LC3B	Beyotime	Cat#C3006
pGADT7-PIK3R4	Youbio	Project No:40068
**SOFTWARES**	**SOURCE**	
Graphpad Prism for statistical analysis	GraphPad	
ImageJ	NIH	
Image Studio	Alias	
Snapgene Viewer	GSL Biotech LLC	
DNAman	LynnonBiosoft	
Odyssey Application Software	Li-cor Biosciences	
Image-Pro Plus software	Media Cybernetics	

Supplementary table 2 RT-PCR primer sequences

Gene	Forward primer	Reverse primer
CD44	CTGCCGCTTTGCAGGTGTA	CATTGTGGGCAAGGTGCTATT
CD44(M)	GATCAACGGTGGCAATGGGACGG	CTCGTCAGCTGTCATACACTGGTCCG
CDKN2A	GACACGCTGGTGGTGCT	GCATGGTTACTGCCTCTGG
CDKN2A(M)	CGCAGGTTCTTGGTCACTGT	TGTTCACGAAAGCCAGAGCG
CDKN1A	ATGAGTTGGGAGGAGGCA	CTGAGCGAGGCACAAGG
CDKN1A(M)	CGAGAACGGTGGAACTTTGAC	CCAGGGCTCAGGTAGACCTT
LC3	ACCAGCACCCCAGCAAA	TCACCAGCAGGAAGAAGGC
LC3(M)	GACCGCTGTAAGGAGGTGC	CTTGACCAACTCGCTCATGTTA
SQSTM1	TACGACTTGTGTAGCGTCTGC	GTGTCCGTGTTTCACCTTCC
ATG14L	GCTATGGAAGGAAAATGGATAACAG	CCGTTGTGCTCGACTGTAAA
BECN1	CAGGATGGTGTCTCTCGCAG	GCATTCCTCACAGAGTGGGT
UVRAG	GGCGTCTTCGACATCTTCGG	GACGGTCTGGCATAATTCCAAA
PIK3C3	GCCTGTAGGAGGAACAACGG	CGATGAGCTTTGGTGAGCTTG
PIK3R4	GAAGATCGGGACTTTTCTGCG	GCCTCGGGAAAGTGTCAAAAT
GAPDH	ACCCACTCCTCCACCTTTG	CTCTTGTGCTCTTGCTGGG
GAPDH(M)	AGGGCCCACTGAAGGGCATCTT	ATTGAGAGCAATGCCAGCCCCG
CLEC-31(C)	CAGCCTCTGTCCACTAGACCT	GGTGAAGCCGAAAGTGCAAC
UNC-51(C)	AAAAGGGCATCGTACATCGT	ATTTTGGGTGCGGGAGTT
EPG-1(C)	ATCCAGCAAATGGAACCAAG	TGGAGTTGATTTTGGAGAATTG
EPG-3(C)	CGAGTGTCAGAGCCTGGATT	CTTTTTGTTGAGGGGCATTG
EPG-9(C)	GAAGTCAGTTGTGAGCAGATTGA	TGGCTTCTTCCACTTCACATT
VPS-34(C)	AACCCTGTCAGAAGGTTGAATC	TGACGAGCAAGTTGAGAGGA
VPS-15(C)	CGATCGATTGAGCACGAG	TGAAGAGCAGGAAGATGTACCA
ATG-16.1(C)	CAGAAGTTGCTTTAGAAGAAAAACG	TTTTGTGTCCTTGTCGGTGA
ATG-7(C)	TCTTGCTATTTCTGCAGTGATGT	GTTCCTGGTCGTGCAACAG
ATG-2(C)	CTCAACCACATGGTGTCGTC	CATCGGTATGGAAAGTAACACCA
ATG-9(C)	GGTCTTCACGATGAGAGTATTATCC	TGCATGTTGAAGCTTGACG
PEG-5(C)	GCGCCAGGATTAGTAGTCAAG	CCAATTGAGGCCAATGAGTT
ACTIN-1(C)	CAAGAGAGGTATCCTTACCCTCAAG	GTCCGGAAGCGTAGAGGGAG
