# Supplementary material for: CD44 connects autophagy decline and ageing in the vascular endothelium
Source: Nat Commun. 2023 Sep 8;14:5524. doi: 10.1038/s41467-023-41346-y (PMC10491636; doi:10.1038/s41467-023-41346-y)
Supplement: Supplementary file 3 — Description of Additional Supplementary Files [file 41467_2023_41346_MOESM3_ESM.pdf]

Description of Additional Supplementary Files

File name: Supplementary Movie 1

Description: The motility of nematodes fed RNAi bacteria targeting empty vector (WT control RNAi) was recorded with a CCD video camera under a stereomicroscope.

File name: Supplementary Movie 2

Description: The motility of nematodes fed RNAi bacteria targeting *clec-31* (*clec-31* RNAi) was recorded with a CCD video camera under a stereomicroscope.
