# Supplementary material for: CD44 connects autophagy decline and ageing in the vascular endothelium
Source: Nat Commun. 2023 Sep 8;14:5524. doi: 10.1038/s41467-023-41346-y (PMC10491636; doi:10.1038/s41467-023-41346-y)
Supplement: Supplementary file 6 — Reporting Summary [file 41467_2023_41346_MOESM6_ESM.pdf]

nature portfolio

Corresponding author(s): Lu Zhang

Last updated by author(s): Aug 22, 2023

Reporting Summary

Nature Portfolio wishes to improve the reproducibility of the work that we publish. This form provides structure for consistency and transparency in reporting. For further information on Nature Portfolio policies, see our Editorial Policies and the Editorial Policy Checklist.

Statistics

For all statistical analyses, confirm that the following items are present in the figure legend, table legend, main text, or Methods section.

n/a	Confirmed	
☐	☒	The exact sample size (*n*) for each experimental group/condition, given as a discrete number and unit of measurement
☐	☒	A statement on whether measurements were taken from distinct samples or whether the same sample was measured repeatedly
☐	☒	The statistical test(s) used AND whether they are one- or two-sided<br>*Only common tests should be described solely by name; describe more complex techniques in the Methods section.*
☒	☐	A description of all covariates tested
☐	☒	A description of any assumptions or corrections, such as tests of normality and adjustment for multiple comparisons
☐	☒	A full description of the statistical parameters including central tendency (e.g. means) or other basic estimates (e.g. regression coefficient) AND variation (e.g. standard deviation) or associated estimates of uncertainty (e.g. confidence intervals)
☐	☒	For null hypothesis testing, the test statistic (e.g. *F*, *t*, *r*) with confidence intervals, effect sizes, degrees of freedom and *P* value noted<br>*Give P values as exact values whenever suitable.*
☒	☐	For Bayesian analysis, information on the choice of priors and Markov chain Monte Carlo settings
☒	☐	For hierarchical and complex designs, identification of the appropriate level for tests and full reporting of outcomes
☒	☐	Estimates of effect sizes (e.g. Cohen's *d*, Pearson's *r*), indicating how they were calculated

Our web collection on statistics for biologists contains articles on many of the points above.

Software and code

Policy information about availability of computer code

Data collection	Li-Cor Odyssey system (Li-cor Biosciences, Cambridge, UK).<br>Eppendorf PCR System (Eppendorf AG, Hamburg, Germany).<br>Confocal laser-scanning microscope (Olympus FLUOVIEW FV3000).<br>Microscope (DM IL LED Fluo, Leica Microsystems CMS GmbH, Wetzlar, Germany).<br>JEM-1230 transmission electron microscopy (JEOL Co., Ltd., Japan).<br>Intelligent noninvasive sphygmomanometer (Softron Biotechnology, BP-2010A)<br>Small animal ultrasound system (VINNO6, LAB)
Data analysis	Fluorescence density: Image J software<br>Immunohistochemical analysis: Image-Pro Plus software<br>Western blot analysis: Image Studio Ver 5.2<br>Statistical analysis: GraphPad Prism 9.0.0<br>Microarray: (1)Single-stranded cDNA was generated from the amplified cRNA with the WT cDNA Synthesis Kit (Affymetrix) and then fragmented and labeled with the WT Terminal Labeling Kit (Affymetrix). (2)Samples were hybridized with GeneChip® Gene1.0 ST Arrays (Affymetrix) and scanned at the UCLA Microarray Core Facility. (3)Array scanning was performed according to the manufacturer's instruction (Affymetrix). (4)Raw data were processed with Affymetrix Gene X.0 ST array Service for background correction and normalization. Genes with > twofold changes and both the false discovery rate (FDR, q) and the Wilcoxon rank-sum test significance level (p) <0.05 were considered significantly regulated.

For manuscripts utilizing custom algorithms or software that are central to the research but not yet described in published literature, software must be made available to editors and reviewers. We strongly encourage code deposition in a community repository (e.g. GitHub). See the Nature Portfolio guidelines for submitting code & software for further information.

Data

Policy information about availability of data

All manuscripts must include a data availability statement. This statement should provide the following information, where applicable:

- Accession codes, unique identifiers, or web links for publicly available datasets
- A description of any restrictions on data availability
- For clinical datasets or third party data, please ensure that the statement adheres to our policy

All data presented in this study are available within the Figures and its supplementary information file. Source data including uncropped western blots and raw microscopy images are provided with this manuscript. The Microarray data have been deposited into the Gene Expression Omnibus database (GEO) in National Center for Biotechnology Information (NCBI) under the accession GSE236523.

Research involving human participants, their data, or biological material

Policy information about studies with human participants or human data. See also policy information about sex, gender (identity/presentation), and sexual orientation and race, ethnicity and racism.

Reporting on sex and gender	N/A
Reporting on race, ethnicity, or other socially relevant groupings	N/A
Population characteristics	N/A
Recruitment	N/A
Ethics oversight	N/A

Note that full information on the approval of the study protocol must also be provided in the manuscript.

Field-specific reporting

Please select the one below that is the best fit for your research. If you are not sure, read the appropriate sections before making your selection.

☒ Life sciences ☐ Behavioural & social sciences ☐ Ecological, evolutionary & environmental sciences

For a reference copy of the document with all sections, see nature.com/documents/nr-reporting-summary-flat.pdf

Life sciences study design

All studies must disclose on these points even when the disclosure is negative.

Sample size	No sample size calculation was performed. Sample size was determined to be sufficient based on the magnitude and consistency of measurable differences between groups. In addition, the sample sizes of this study were determined on the basis of similar published studies. In vitro experiments, the sample size for each group was greater than 5. Because the reduction of experimental animals is an important principle for animal experiments, the invivo studies were Use as few animals as possible under precursors that ensure experimental rigor which also reached statistical significance (p<0.05),between experimental groups. Meanwhile, sample sizes for the in vivo experiments are similar to those generally employed and accepted in the field (Nat. Commun. 8, 146 (2017); Nat. Med. 18, 1077 (2012)) and are adequate to support our conclusions with statistical significance.
Data exclusions	None.
Replication	Experimental results were reproducible. Replicate numbers are indicated in the figure legends.
Randomization	Samples were randomly allocated to corresponding experimental groups. Cells were cultured and maintained in the same environment and randomly allocated to each group.For invivo study,Male and female mice were randomly assigned to different groups.
Blinding	In all experiments,Investigator was blinded to the different groups at the time of measurement.

Behavioural & social sciences study design

All studies must disclose on these points even when the disclosure is negative.

Study description	*Briefly describe the study type including whether data are quantitative, qualitative, or mixed-methods (e.g. qualitative cross-sectional, quantitative experimental, mixed-methods case study).*
Research sample	*State the research sample (e.g. Harvard university undergraduates, villagers in rural India) and provide relevant demographic information (e.g. age, sex) and indicate whether the sample is representative. Provide a rationale for the study sample chosen. For studies involving existing datasets, please describe the dataset and source.*
Sampling strategy	*Describe the sampling procedure (e.g. random, snowball, stratified, convenience). Describe the statistical methods that were used to predetermine sample size OR if no sample-size calculation was performed, describe how sample sizes were chosen and provide a rationale for why these sample sizes are sufficient. For qualitative data, please indicate whether data saturation was considered, and what criteria were used to decide that no further sampling was needed.*
Data collection	*Provide details about the data collection procedure, including the instruments or devices used to record the data (e.g. pen and paper, computer, eye tracker, video or audio equipment) whether anyone was present besides the participant(s) and the researcher, and whether the researcher was blind to experimental condition and/or the study hypothesis during data collection.*
Timing	*Indicate the start and stop dates of data collection. If there is a gap between collection periods, state the dates for each sample cohort.*
Data exclusions	*If no data were excluded from the analyses, state so OR if data were excluded, provide the exact number of exclusions and the rationale behind them, indicating whether exclusion criteria were pre-established.*
Non-participation	*State how many participants dropped out/declined participation and the reason(s) given OR provide response rate OR state that no participants dropped out/declined participation.*
Randomization	*If participants were not allocated into experimental groups, state so OR describe how participants were allocated to groups, and if allocation was not random, describe how covariates were controlled.*

Ecological, evolutionary & environmental sciences study design

All studies must disclose on these points even when the disclosure is negative.

Study description	*Briefly describe the study. For quantitative data include treatment factors and interactions, design structure (e.g. factorial, nested, hierarchical), nature and number of experimental units and replicates.*
Research sample	*Describe the research sample (e.g. a group of tagged Passer domesticus, all Stenocereus thurberi within Organ Pipe Cactus National Monument), and provide a rationale for the sample choice. When relevant, describe the organism taxa, source, sex, age range and any manipulations. State what population the sample is meant to represent when applicable. For studies involving existing datasets, describe the data and its source.*
Sampling strategy	*Note the sampling procedure. Describe the statistical methods that were used to predetermine sample size OR if no sample-size calculation was performed, describe how sample sizes were chosen and provide a rationale for why these sample sizes are sufficient.*
Data collection	*Describe the data collection procedure, including who recorded the data and how.*
Timing and spatial scale	*Indicate the start and stop dates of data collection, noting the frequency and periodicity of sampling and providing a rationale for these choices. If there is a gap between collection periods, state the dates for each sample cohort. Specify the spatial scale from which the data are taken*
Data exclusions	*If no data were excluded from the analyses, state so OR if data were excluded, describe the exclusions and the rationale behind them, indicating whether exclusion criteria were pre-established.*
Reproducibility	*Describe the measures taken to verify the reproducibility of experimental findings. For each experiment, note whether any attempts to repeat the experiment failed OR state that all attempts to repeat the experiment were successful.*
Randomization	*Describe how samples/organisms/participants were allocated into groups. If allocation was not random, describe how covariates were controlled. If this is not relevant to your study, explain why.*
Blinding	*Describe the extent of blinding used during data acquisition and analysis. If blinding was not possible, describe why OR explain why blinding was not relevant to your study.*

Did the study involve field work? ☐ Yes ☐ No

Field work, collection and transport

Field conditions	*Describe the study conditions for field work, providing relevant parameters (e.g. temperature, rainfall).*
Location	*State the location of the sampling or experiment, providing relevant parameters (e.g. latitude and longitude, elevation, water depth).*
Access & import/export	*Describe the efforts you have made to access habitats and to collect and import/export your samples in a responsible manner and in compliance with local, national and international laws, noting any permits that were obtained (give the name of the issuing authority, the date of issue, and any identifying information).*
Disturbance	*Describe any disturbance caused by the study and how it was minimized.*

Reporting for specific materials, systems and methods

We require information from authors about some types of materials, experimental systems and methods used in many studies. Here, indicate whether each material, system or method listed is relevant to your study. If you are not sure if a list item applies to your research, read the appropriate section before selecting a response.

Materials & experimental systems

n/a	Involved in the study	
☐	☒	Antibodies
☐	☒	Eukaryotic cell lines
☒	☐	Palaeontology and archaeology
☐	☒	Animals and other organisms
☒	☐	Clinical data
☒	☐	Dual use research of concern
☒	☐	Plants

Methods

n/a	Involved in the study	
☒	☐	ChIP-seq
☒	☐	Flow cytometry
☒	☐	MRI-based neuroimaging

Antibodies

Antibodies used

For WB:
Mouse mAb to CD44 (Cell Signaling Technology, #5640, clone (8E2), lot:4(m), dilution 1:1000);
Rabbit mAb to p16 (ABcam, #ab51243, clone (EP1551Y), lot:GR3227034-14, dilution 1:1000);
Rabbit mAb to p21 (ABcam, Cat#ab109199, clone(EPR3993), lot:1, dilution 1:1000);
Mouse mAb to LC3 (SIGMA, #SAB1305552, clone (APG8), lot:SG150518AA, dilution 1:15000);
Rabbit mAb to LC3 (Cell Signaling Technology, #12741S, clone (D3U4C), lot:5 ,dilution 1:1000);
Rabbit pAb to LC3 (Proteintech, #14600-1-AP, lot:00109445, dilution 1:1000);
Rabbit mAb to Anti-p62/SQSTM1 (SIGMA, #P0067, lot:055M4816V, dilution 1:15000);
Rabbit pAb to DDK (OriGene, #TA100023, lot:AP6170, dilution 1:1000);
Rabbit pAb to FLAG (Proteintech, #20543-1-AP, clone (FLAG tag epitope), lot:00079983, dilution 1:2000);
Rabbit mAb to DYKDDDDK Tag (Cell Signaling Technology, #14793, clone (Binds to same epitope as Sigma's Anti-FLAG® M2 Antibody), lot:7 , dilution 1:1000);
Rabbit mAb to PIK3R4 antibody produced (SIGMA, HPA036032, clone lot:R32822, dilution 1:1000);
Rabbit mAb to VPS34 antibody produced (SIGMA, #V9764, clone lot:046M4850V, dilution 1:1000);
Rabbit mAb to Atg14 (Cell Signaling Technology, #96752, clone (D1A1N) lot:1, dilution 1:1000);
Rabbit mAb to Beclin 1 (SIGMA, #B6061, dilution 1:1000);
Rabbit pAb to Beclin 1 (bioworld, #AP6020, lot:AAD12162, dilution 1:2000);
Mouse mAb to UVRAG (SIGMA, #SAB4200005, lot:099K4809, dilution 1:1000);
Rabbit mAb to UVRAG (Cell Signaling Technology, #13115, clone (D2Q1Z) lot:1,dilution 1:1000);
Rabbit mAb to UVRAG (Cell Signaling Technology #5320, lot:2,dilution 1:1000);
Mouse mAb to Stat3 (124H6) (Cell Signaling Technology #9139, clone (124H6) lot:12,dilution 1:1000);
Rabbit mAb to Stat3 (D3Z2G) (Cell Signaling Technology #12640,clone(D3Z2G) lot:4, dilution 1:1000);
Rabbit mAb to P-Stat3 (Tyr705) (Cell Signaling Technology #9145,clone (D3A7) lot:31,dilution 1:20000);
Rabbit pAb to ULK1 (ABclonal, #A8529, dilution 1:1000)
Rabbit pAb to Atg13 (ABclonal, #A0690, dilution 1:1000)
Rabbit pAb to FIP200 (ABclonal, #A14685, dilution 1:1000)
Rabbit mAb to P-Stat1 (Y701) (ABclonal, #AP0045, dilution 1:1000)
Rabbit pAb to Stat1 (ABclonal, #A12075, dilution 1:1000)
Rabbit pAb to Atg101 (Proteintech, #26562-1-AP, dilution 1:2000)
For IF:
Rabbit mAb to CD44 (Cell Signaling Technology, #3578, clone (Leu135), lot:2(R) , dilution 1:300);
Rabbit mAb to p16 (ABcam, #ab51243, clone (EP1551Y), lot:GR3227034-14, dilution 1:300);
Rabbit mAb to p21 (ABclonal, #A1483, dilution 1:500);
Rabbit mAb to p21(Cell Signaling Technology, #37543, clone (E2R7A) lot,dilution 1:500);
Rabbit mAb to Anti-LC3B ?SIGMA, #L7543,dilution 1:300);

Rabbit mAb to Anti-p62/SQSTM1 (SIGMA, #P0067, lot:055M4816V, dilution 1:500);
Rabbit mAb to PIK3R4 antibody produced (SIGMA, HPA036032, clone lot:R32822, dilution 1:500);
Rabbit mAb to VPS34 antibody produced (SIGMA, #V9764, clone lot:046M4850V, dilution 1:300);
Rabbit mAb to Atg14 (Cell Signaling Technology, #96752, clone (D1A1N) lot:1, dilution 1:300);
Rabbit mAb to Beclin 1 (SIGMA, #B6061, dilution 1:300);
Rabbit mAb to UVRAG (Cell Signaling Technology, #13115, clone (D2Q1Z) lot:1,dilution 1:300);
Rabbit mAb to Stat3 (D3Z2G) (Cell Signaling Technology #12640,clone(D3Z2G) lot:4, dilution 1:300)
Mouse mAb to DDDDK-Tag (ABclonal, #AE005, dilution 1:200)
Goat Anti-Rabbit IgG H&L (Alexa Fluor® 647) (Abcam, #ab150079, dilution 1:200)
Goat Anti-Mouse IgG H&L (Alexa Fluor® 488) (Abcam, #ab150113, dilution 1:200)
Mouse pAb to CD31 (Santa cruz, #sc-376764, dilution 1:500)
For IHC:
Rabbit mAb to CD44 (Cell Signaling Technology, #3578, clone (Leu135), lot:2(R) , dilution 1:200);
Rabbit mAb to p16 (ABcam, #ab51243, clone (EP1551Y), lot:GR3227034-14, dilution 1:200);
Rabbit mAb to p21 (ABclonal, #A1483, dilution 1:200);
Rabbit mAb to Anti-LC3B (SIGMA, #L7543,dilution 1:200);
Rabbit pAb to p-eNOS (ABclonal, #AP0515, dilution 1:200);
Rabbit mAb to Anti-p62/SQSTM1 (SIGMA, #P0067, lot:055M4816V, dilution 1:200);

Validation

All antibodies used in this paper were validated by WB/IHC/IF in published articles, or in reference to manufacturer's websites, or in relevant knockout mice.
1.Mouse mAb to CD44 (Cell Signaling Technology, #5640, clone (8E2), lot:4(m), dilution 1:1000);
https://www.cellsignal.cn/products/primary-antibodies/cd44-8e2-mouse-mab/5640?site-search-type=Products&N=4294956287&Ntt=5640&fromPage=plp&_requestid=2570715
2.Rabbit mAb to CD44 (Cell Signaling Technology, #3578, clone (Leu135), lot:2(R) , dilution 1:1000);
https://www.cellsignal.cn/products/primary-antibodies/cd44-antibody/3578?site-search-type=Products&N=4294956287&Ntt=3578&fromPage=plp&_requestid=2571161
3.Rabbit mAb to p16 (ABcam, #ab51243, clone (EP1551Y), lot:GR3227034-14, dilution 1:1000);
https://www.abcam.cn/products/primary-antibodies/arpc5p16-arc-antibody-ep1551y-ab51243.html
4.Rabbit mAb to p21 (ABcam, #ab109199, clone(EPR3993), lot:1, dilution 1:1000);
https://www.abcam.cn/products/primary-antibodies/p21-antibody-epr3993-ab109199.html
5.Rabbit mAb to p21 (ABclonal, #A1483, dilution 1:500);
https://abclonal.com.cn/catalog/A1483
6.Rabbit mAb to p21(Cell Signaling Technology, #37543, clone (E2R7A) lot,dilution 1:500);
https://www.cellsignal.cn/products/primary-antibodies/p21-waf1-cip1-e2r7a-rabbit-mab/37543?site-search-type=Products&N=4294956287&Ntt=37543&fromPage=plp&_requestid=2571449
7.Mouse mAb to LC3 (SIGMA, #SAB1305552, clone (APG8), lot:SG150518AA, dilution 1:15000);
https://www.sigmaaldrich.cn/CN/zh/search/sab1305552?focus=products&page=1&perpage=30&sort=relevance&term=sab1305552&type=product
8.Rabbit mAb to Anti-LC3B SIGMA, #L7543,dilution 1:1000);
https://www.sigmaaldrich.cn/CN/zh/search/l7543?focus=products&page=1&perpage=30&sort=relevance&term=l7543&type=product
9.Rabbit mAb to LC3 (Cell Signaling Technology, #12741S, clone (D3U4C), lot:5 ,dilution 1:1000);
https://www.cellsignal.cn/products/primary-antibodies/lc3a-b-d3u4c-xp-rabbit-mab/12741?site-search-type=Products&N=4294956287&Ntt=12741s&fromPage=plp&_requestid=2571715
10.Rabbit pAb to LC3 (Proteintech, #14600-1-AP, lot:00109445, dilution 1:1000);
https://www.ptgcn.com/products/MAP1LC3B-Antibody-14600-1-AP.htm
11.Rabbit pAb to p-eNOS (ABclonal, #AP0515, dilution 1:500);
https://abclonal.com.cn/catalog/AP0515
12.Rabbit mAb to Anti-p62/SQSTM1 (SIGMA, #P0067, lot:055M4816V, dilution 1:15000);
https://www.sigmaaldrich.cn/CN/zh/search/p0067?focus=products&page=1&perpage=30&sort=relevance&term=p0067&type=product
13.Rabbit pAb to DDK (OriGene, #TA100023, lot:AP6170, dilution 1:1000);
https://www.origene.com.cn/search?q=TA100023
14.Rabbit pAb to FLAG (Proteintech, #20543-1-AP, clone (FLAG tag epitope), lot:00079983, dilution 1:2000);
https://www.ptgcn.com/products/Flag-Tag-Antibody-20543-1-AP.htm
15.Rabbit mAb to DYKDDDDK Tag (Cell Signaling Technology, #14793, clone (Binds to same epitope as Sigma's Anti-FLAG® M2 Antibody), lot:7 , dilution 1:1000);
https://www.cellsignal.cn/products/primary-antibodies/dykddddk-tag-d6w5b-rabbit-mab-binds-to-same-epitope-as-sigma-s-anti-flag-m2-antibody/14793?site-search-type=Products&N=4294956287&Ntt=14793&fromPage=plp&_requestid=2572114
16.Rabbit mAb to PIK3R4 antibody produced (SIGMA, #HPA036032, clone lot:R32822, dilution 1:1000);
https://www.sigmaaldrich.cn/CN/zh/search/hpa036032?focus=products&page=1&perpage=30&sort=relevance&term=hpa036032&type=product
17.Rabbit mAb to VPS34 antibody produced (SIGMA, #V9764, clone lot:046M4850V, dilution 1:1000);
https://www.sigmaaldrich.cn/CN/zh/search/v9764?focus=products&page=1&perpage=30&sort=relevance&term=v9764&type=product
18.Rabbit mAb to Atg14 (Cell Signaling Technology, #96752, clone (D1A1N) lot:1, dilution 1:1000);
https://www.cellsignal.cn/products/primary-antibodies/atg14-d1a1n-rabbit-mab/96752?site-search-type=Products&N=4294956287&Ntt=96752&fromPage=plp&_requestid=2572283
19.Rabbit mAb to Beclin 1 (SIGMA, #B6061, dilution 1:1000);
https://www.sigmaaldrich.cn/CN/zh/search/b6061?focus=products&page=1&perpage=30&sort=relevance&term=b6061&type=product
20.Rabbit pAb to Beclin 1 (bioworld, #AP6020, lot:AAD12162, dilution 1:2000);
https://www.bioworlde.com
21.Mouse mAb to UVRAG (SIGMA, #SAB4200005, lot:099K4809, dilution 1:1000);
https://www.sigmaaldrich.cn/CN/zh/search/sab4200005?

focus=products&page=1&perpage=30&sort=relevance&term=sab4200005&type=product
22.Rabbit mAb to UVRAG (Cell Signaling Technology, #13115, clone (D2Q1Z) lot:1,dilution 1:1000);
https://www.cellsignal.cn/products/primary-antibodies/uvrag-d2q1z-rabbit-mab/13115?site-search-type=Products&N=4294956287&Ntt=13115&fromPage=plp&_requestid=2572689
23.Rabbit mAb to UVRAG (Cell Signaling Technology #5320, lot:2,dilution 1:1000);
https://www.cellsignal.cn/products/primary-antibodies/uvrag-antibody/5320?site-search-type=Products&N=4294956287&Ntt=5320&fromPage=plp
24.Mouse mAb to Stat3 (124H6) (Cell Signaling Technology #9139, clone (124H6) lot:12,dilution 1:1000);
https://www.cellsignal.cn/products/primary-antibodies/stat3-124h6-mouse-mab/9139?site-search-type=Products&N=4294956287&Ntt=9139&fromPage=plp&_requestid=2572830
25.Rabbit mAb to Stat3 (D3Z2G) (Cell Signaling Technology #12640,clone(D3Z2G) lot:4, dilution 1:1000);
https://www.cellsignal.cn/products/primary-antibodies/stat3-d3z2g-rabbit-mab/12640?site-search-type=Products&N=4294956287&Ntt=12640&fromPage=plp&_requestid=2572873
26.Rabbit mAb to P-Stat3 (Tyr705) (Cell Signaling Technology #9145,clone (D3A7) lot:31,dilution 1:20000);
https://www.cellsignal.cn/products/primary-antibodies/phospho-stat3-tyr705-d3a7-xp-rabbit-mab/9145?site-search-type=Products&N=4294956287&Ntt=9145&fromPage=plp&_requestid=2572937
27.Rabbit pAb to ULK1 (ABclonal, #A8529, dilution 1:1000)
https://abclonal.com.cn/catalog/A8529
28.Rabbit pAb to Atg13 (ABclonal, #A0690, dilution 1:1000)
https://abclonal.com.cn/catalog/A0690
29.Rabbit pAb to FIP200 (ABclonal, #A14685, dilution 1:1000)
https://abclonal.com.cn/catalog/A14685
30.Rabbit mAb to P-Stat1 (Y701) (ABclonal, #AP0045, dilution 1:1000)
https://abclonal.com.cn/catalog/AP0045
31.Rabbit pAb to Stat1 (ABclonal, #A12075, dilution 1:1000)
https://abclonal.com.cn/catalog/A12075
32.Rabbit pAb to Atg101 (Proteintech, #26562-1-AP, dilution 1:2000)
https://www.ptgcn.com/products/ATG101-Antibody-26562-1-AP.htm
33.Goat Anti-Rabbit IgG H&L (Alexa Fluor® 647) (Abcam, #ab150079, dilution 1:200)
https://www.abcam.cn/products/secondary-antibodies/goat-rabbit-igg-hl-alexa-fluor-647-ab150079.html
34.Goat Anti-Mouse IgG H&L (Alexa Fluor® 488) (Abcam, #ab150113, dilution 1:200)
https://www.abcam.cn/products/secondary-antibodies/goat-mouse-igg-hl-alexa-fluor-488-ab150113.html
35.Mouse pAb to CD31 (Santa cruz, #sc-376764, dilution 1:500)
https://www.scbt.com/p/pecam-1-antibody-h-3?requestFrom=search
36.Mouse mAb to DDDDK-Tag (ABclonal, #AE005, dilution 1:200)
https://abclonal.com.cn/catalog/AE005

Eukaryotic cell lines

Policy information about cell lines and Sex and Gender in Research

Cell line source(s)

HUVECs were isolated from the vein of the umbilical cords of healthy pregnant women in our lab. HEK293T cells were purchased from the American Type Culture Collection (ATCC). AH109 Yeast Strain were purchased from the Shanghai Weidi Biotechnology.

Authentication

HUVECs were authentication based on STR fingerprinting before use. All other cell lines were authenticated by the vendor.

Mycoplasma contamination

Cell lines were not tested for mycoplasma contamination.

Commonly misidentified lines
(See ICLAC register)

No commonly misidentified cell lines were used in the study.

Palaeontology and Archaeology

Specimen provenance

Provide provenance information for specimens and describe permits that were obtained for the work (including the name of the issuing authority, the date of issue, and any identifying information). Permits should encompass collection and, where applicable, export.

Specimen deposition

Indicate where the specimens have been deposited to permit free access by other researchers.

Dating methods

If new dates are provided, describe how they were obtained (e.g. collection, storage, sample pretreatment and measurement), where they were obtained (i.e. lab name), the calibration program and the protocol for quality assurance OR state that no new dates are provided.

☐ Tick this box to confirm that the raw and calibrated dates are available in the paper or in Supplementary Information.

Ethics oversight

Identify the organization(s) that approved or provided guidance on the study protocol, OR state that no ethical approval or guidance was required and explain why not.

Note that full information on the approval of the study protocol must also be provided in the manuscript.

Animals and other research organisms

Policy information about studies involving animals; ARRIVE guidelines recommended for reporting animal research, and Sex and Gender in Research

Laboratory animals	Mice:C57BL/6 mice (NO.11400700255206), B6J.Cg-Gt(ROSA)26Sorem16(CAG-Cd44icd)/J mice (NO SO171215JW2), B6.Cg-Tg(Tek-cre)12Flv/J mice (Stock No:C001001), B6.129(Cg)-Cd44tm1Hbg/J mice (Stock No: 005085), were used in this study. Caenorhabditis elegans N2(WT) (this paper), DA2123:adls2122(lgg-1p::gfp::lgg-1) (this paper), CLEC-31 OE,(Project No:ZLUO1-02). All mice were cultured in suitable temperature and humidity environment with ad libitum access to water and food (25?, suitable humidity (typically 50%), 12 hours dark/light cycle).<br>Fig 1: For d : WT mice, young (2-4 months), middled-aged (8-12 months), and aged (≥ 19 months). For e : WT mice,young (2-4 months), middled-aged (8-12 months), and aged (≥ 19 months). For f:WT mice, young (2-4 months), middled-aged (8-12 months), and aged (≥ 19 months). For g : WT mice, young (2-4 months), middled-aged (8-12 months), and aged (≥ 19 months). For l, m, n, r : WT and CD44 KO mice, (16-19 months).For o, p, q, s: WT and CD44 KO mice, (16-19 months).<br>Fig 2: For a, b : WT and CD44 KO mice, (16-19 months).<br>Fig 3: For i, j, k : WT and CD44EC KI mice, (8-10 months).<br>Fig 7:For a, b, c, d, e, f : WT and CD44EC KI mice, (8-10 months). For j, h, i : WT and CD44EC KI mice, (14-17 months). For l, m, n : CD44EC KI mice, (8 months).<br>Supplementary fig 1:For a, c, d, e : WT and CD44 KO mice, (2-4 months). For b :WT and CD44 KO mice, (4 months).<br>Supplementary fig 8:For a, b, c, d : WT and CD44EC KI mice, (8-10 months).<br>Supplementary fig 9:WT and CD44EC KI mice, (14-17 months).<br>Supplementary fig 11:For b, c : CD44EC KI mice, (8 months).
Wild animals	This study did not involve the wild animals.
Reporting on sex	In this study, both males and females were present, and the number of males and females in the study design group was roughly similar.
Field-collected samples	This study did not include field-collected samples.
Ethics oversight	All studies with mice were approved by Cyagen Biological Laboratory Animal Center Animal Care Committee (ETHICS number: ACU22-035).

Note that full information on the approval of the study protocol must also be provided in the manuscript.

Clinical data

Policy information about clinical studies

All manuscripts should comply with the ICMJE guidelines for publication of clinical research and a completed CONSORT checklist must be included with all submissions.

Clinical trial registration	*Provide the trial registration number from ClinicalTrials.gov or an equivalent agency.*
Study protocol	*Note where the full trial protocol can be accessed OR if not available, explain why.*
Data collection	*Describe the settings and locales of data collection, noting the time periods of recruitment and data collection.*
Outcomes	*Describe how you pre-defined primary and secondary outcome measures and how you assessed these measures.*

Dual use research of concern

Policy information about dual use research of concern

Hazards

Could the accidental, deliberate or reckless misuse of agents or technologies generated in the work, or the application of information presented in the manuscript, pose a threat to:

No	Yes	
☒	☐	Public health
☒	☐	National security
☒	☐	Crops and/or livestock
☒	☐	Ecosystems
☒	☐	Any other significant area

Experiments of concern

Does the work involve any of these experiments of concern:

No	Yes	
☒	☐	Demonstrate how to render a vaccine ineffective
☒	☐	Confer resistance to therapeutically useful antibiotics or antiviral agents
☒	☐	Enhance the virulence of a pathogen or render a nonpathogen virulent
☒	☐	Increase transmissibility of a pathogen
☒	☐	Alter the host range of a pathogen
☒	☐	Enable evasion of diagnostic/detection modalities
☒	☐	Enable the weaponization of a biological agent or toxin
☒	☐	Any other potentially harmful combination of experiments and agents

Plants

Seed stocks	*Report on the source of all seed stocks or other plant material used. If applicable, state the seed stock centre and catalogue number. If plant specimens were collected from the field, describe the collection location, date and sampling procedures.*
Novel plant genotypes	*Describe the methods by which all novel plant genotypes were produced. This includes those generated by transgenic approaches, gene editing, chemical/radiation-based mutagenesis and hybridization. For transgenic lines, describe the transformation method, the number of independent lines analyzed and the generation upon which experiments were performed. For gene-edited lines, describe the editor used, the endogenous sequence targeted for editing, the targeting guide RNA sequence (if applicable) and how the editor was applied.*
Authentication	*Describe any authentication procedures for each seed stock used or novel genotype generated. Describe any experiments used to assess the effect of a mutation and, where applicable, how potential secondary effects (e.g. second site T-DNA insertions, mosiacism, off-target gene editing) were examined.*

ChIP-seq

Data deposition

☐ Confirm that both raw and final processed data have been deposited in a public database such as GEO.

☐ Confirm that you have deposited or provided access to graph files (e.g. BED files) for the called peaks.

Data access links<br>*May remain private before publication.*	*For "Initial submission" or "Revised version" documents, provide reviewer access links. For your "Final submission" document, provide a link to the deposited data.*
Files in database submission	*Provide a list of all files available in the database submission.*
Genome browser session<br>(e.g. UCSC)	*Provide a link to an anonymized genome browser session for "Initial submission" and "Revised version" documents only, to enable peer review. Write "no longer applicable" for "Final submission" documents.*

Methodology

Replicates	*Describe the experimental replicates, specifying number, type and replicate agreement.*
Sequencing depth	*Describe the sequencing depth for each experiment, providing the total number of reads, uniquely mapped reads, length of reads and whether they were paired- or single-end.*
Antibodies	*Describe the antibodies used for the ChIP-seq experiments; as applicable, provide supplier name, catalog number, clone name, and lot number.*
Peak calling parameters	*Specify the command line program and parameters used for read mapping and peak calling, including the ChIP, control and index files used.*
Data quality	*Describe the methods used to ensure data quality in full detail, including how many peaks are at FDR 5% and above 5-fold enrichment.*
Software	*Describe the software used to collect and analyze the ChIP-seq data. For custom code that has been deposited into a community repository, provide accession details.*

Flow Cytometry

Plots

Confirm that:

- [ ] The axis labels state the marker and fluorochrome used (e.g. CD4-FITC).
- [ ] The axis scales are clearly visible. Include numbers along axes only for bottom left plot of group (a 'group' is an analysis of identical markers).
- [ ] All plots are contour plots with outliers or pseudocolor plots.
- [ ] A numerical value for number of cells or percentage (with statistics) is provided.

Methodology

Sample preparation	*Describe the sample preparation, detailing the biological source of the cells and any tissue processing steps used.*
Instrument	*Identify the instrument used for data collection, specifying make and model number.*
Software	*Describe the software used to collect and analyze the flow cytometry data. For custom code that has been deposited into a community repository, provide accession details.*
Cell population abundance	*Describe the abundance of the relevant cell populations within post-sort fractions, providing details on the purity of the samples and how it was determined.*
Gating strategy	*Describe the gating strategy used for all relevant experiments, specifying the preliminary FSC/SSC gates of the starting cell population, indicating where boundaries between "positive" and "negative" staining cell populations are defined.*

- [ ] Tick this box to confirm that a figure exemplifying the gating strategy is provided in the Supplementary Information.

Magnetic resonance imaging

Experimental design

Design type	*Indicate task or resting state; event-related or block design.*
Design specifications	*Specify the number of blocks, trials or experimental units per session and/or subject, and specify the length of each trial or block (if trials are blocked) and interval between trials.*
Behavioral performance measures	*State number and/or type of variables recorded (e.g. correct button press, response time) and what statistics were used to establish that the subjects were performing the task as expected (e.g. mean, range, and/or standard deviation across subjects).*

Acquisition

Imaging type(s)	*Specify: functional, structural, diffusion, perfusion.*
Field strength	*Specify in Tesla*
Sequence & imaging parameters	*Specify the pulse sequence type (gradient echo, spin echo, etc.), imaging type (EPI, spiral, etc.), field of view, matrix size, slice thickness, orientation and TE/TR/flip angle.*
Area of acquisition	*State whether a whole brain scan was used OR define the area of acquisition, describing how the region was determined.*

Diffusion MRI ☐ Used ☐ Not used

Preprocessing

Preprocessing software	*Provide detail on software version and revision number and on specific parameters (model/functions, brain extraction, segmentation, smoothing kernel size, etc.).*
Normalization	*If data were normalized/standardized, describe the approach(es): specify linear or non-linear and define image types used for transformation OR indicate that data were not normalized and explain rationale for lack of normalization.*
Normalization template	*Describe the template used for normalization/transformation, specifying subject space or group standardized space (e.g. original Talairach, MNI305, ICBM152) OR indicate that the data were not normalized.*
Noise and artifact removal	*Describe your procedure(s) for artifact and structured noise removal, specifying motion parameters, tissue signals and physiological signals (heart rate, respiration).*

Volume censoring | *Define your software and/or method and criteria for volume censoring, and state the extent of such censoring.*

Statistical modeling & inference

Model type and settings | *Specify type (mass univariate, multivariate, RSA, predictive, etc.) and describe essential details of the model at the first and second levels (e.g. fixed, random or mixed effects; drift or auto-correlation).*

Effect(s) tested | *Define precise effect in terms of the task or stimulus conditions instead of psychological concepts and indicate whether ANOVA or factorial designs were used.*

Specify type of analysis: ☐ Whole brain ☐ ROI-based ☐ Both

Statistic type for inference | *Specify voxel-wise or cluster-wise and report all relevant parameters for cluster-wise methods.*

(See Eklund et al. 2016)

Correction | *Describe the type of correction and how it is obtained for multiple comparisons (e.g. FWE, FDR, permutation or Monte Carlo).*

Models & analysis

n/a	Involved in the study
☐	☐ Functional and/or effective connectivity
☐	☐ Graph analysis
☐	☐ Multivariate modeling or predictive analysis

Functional and/or effective connectivity | *Report the measures of dependence used and the model details (e.g. Pearson correlation, partial correlation, mutual information).*

Graph analysis | *Report the dependent variable and connectivity measure, specifying weighted graph or binarized graph, subject- or group-level, and the global and/or node summaries used (e.g. clustering coefficient, efficiency, etc.).*

Multivariate modeling and predictive analysis | *Specify independent variables, features extraction and dimension reduction, model, training and evaluation metrics.*
